# Regulation of selective class switching provides long-term therapeutic benefits for hay fever

**DOI:** 10.1172/jci.insight.190240

**Published:** 2025-10-21

**Authors:** Naoki Morita, Kohta Yamamoto, Ryutaro Tamano, Peng Gao, Takahiro Nagatake, Takenori Inomata, Tianxiang Huang, Yasuhiro Yamada, Takahiro Adachi, Manabu Sugai, Keiichi I. Nakayama, Hirotatsu Kojima, Reiko Shinkura

**Affiliations:** 1 Laboratory of Immunology and Infection Control, Institute for Quantitative Biosciences, The University of Tokyo, Tokyo, Japan.; 2 Laboratory of Applied Immunology, Graduate School of Biological Sciences, Nara Institute of Science and Technology, Nara, Japan.; 3 Laboratory of Functional Anatomy, Department of Life Sciences, School of Agriculture, Meiji University, Kanagawa, Japan.; 4 Department of Ophthalmology,; 5 Department of Hospital Administration, and; 6 Department of Telemedicine and Mobile Health, Juntendo University Graduate School of Medicine, Tokyo, Japan.; 7 Department of Molecular Pathology, Graduate School of Medicine, The University of Tokyo, Tokyo, Japan.; 8 Department of Immunology, Medical Research Institute, Tokyo Medical and Dental University, Tokyo, Japan.; 9 Advanced Research Institute, Tokyo Medical and Dental University, Tokyo, Japan.; 10 Department of Molecular Genetics, Division of Medicine, Faculty of Medical Sciences, and; 11 Life Science Innovation Center, University of Fukui, Fukui, Japan.; 12 Anticancer Strategies Laboratory, TMDU Advanced Research Institute, Tokyo Medical and Dental University, Tokyo, Japan.; 13 Drug Discovery Initiative, Graduate School of Pharmaceutical Sciences,; 14 Graduate School of Frontier Science, and; 15 Collaborative Research Institute for Innovative Microbiology, The University of Tokyo, Tokyo, Japan.

**Keywords:** Immunology, Inflammation, Adaptive immunity, Allergy, Immunoglobulins

## Abstract

IgA protects the body from invaders in the mucosal sites, but its role in allergic diseases, such as hay fever, is poorly understood. We demonstrate an increased susceptibility to cedar-pollen-induced hay fever associated with increasing pollen penetration into the body in IgA-deficient mice, indicating that IgA prevents pollen invasion in the mucosa. We identified bryostatin 1, an anticarcinogenic protein kinase Cδ (PKCδ) activator, as an IgA/IgE class-switching regulator in B cells. Bryostatin 1 enhanced IgA production through induction of germline transcript α (GLTα) via the PKCδ/MEK/ERK/RUNX1 pathway and suppressed IgE by reducing GLTε through the PKCδ/STAT5/ID2 pathway. Production of Th2 cytokines and eosinophil infiltration in the lungs was also reduced. Furthermore, hay fever alleviation by bryostatin 1 demonstrated diminished symptoms in mice in vivo 3 months subsequent to intranasal administration.

## Abstract 2

Activation of PKCδ ameliorates hay fever through prevention of pollen penetration by enhancement of IgA production by regulation of class-switching in B cells.

## Graphical abstract

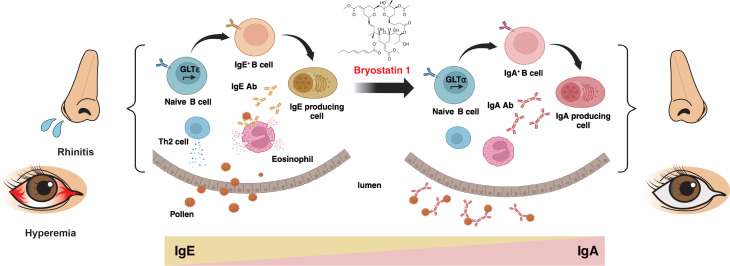

## Introduction

IgA is the most dominant antibody isotype in mammals and is mainly secreted in mucosal sites to protect against penetration of invaders from the outside (1, 2). IgA antibodies are produced by IgA-producing cells that differentiate from IgM^+^ B cells via IgA^+^ B cells through class switching (3, 4). Sterile germline transcript α (GLTα) and activation-induced cytidine deaminase (AID) facilitate recombination between IgM and IgA switch region sequences and subsequently generate the IgA heavy chain without changing the variable region of the antibody (3, 5, 6). Expression of specific spliced GLT determines the direction of class switching from IgM to the corresponding isotype. Selective IgA deficiency is a primary congenital immunodeficiency. Half of those patients have a variety of diseases, including allergic disorders (7–9). However, it remains unclear how IgA regulates allergic diseases.

Although the number of patients with allergic diseases, including pollen hay fever, has increased, the development of effective therapeutic medicines to treat it is still lacking (10). Hay fever is an overreactive immune response to allergens that is associated with symptoms in the mucosa such as rhinitis, conjunctivitis, and lung inflammation. These allergies are mainly caused by helper type II T cell–related (Th2-related) cytokines and IgE antibodies (11–14). Allergen-specific IgE induces degranulation to secrete chemical mediators from eosinophils, mast cells, and basophils by cross-linking of the Fcε receptor with the allergen-bound IgE antibodies (11, 12). At present, only symptomatic or desensitization therapies are available for treatment of hay fever (11, 15–17). Therefore, there is a demand for the development of novel curative treatments.

Here, we propose what we believe is a novel therapeutic strategy for hay fever that shifts antibody production from IgE to IgA by regulating class switching via PKCδ activation. This strategy reduces allergen-specific IgE, while enhancing production of allergen-specific IgA at the mucosa to protect against allergen penetration. Our work uncovered the molecular mechanism for the selective regulation of IgA and IgE class switching.

## Results

### IgA deficiency causes a high susceptibility to cedar-pollen-induced hay fever.

**Figure 1:**
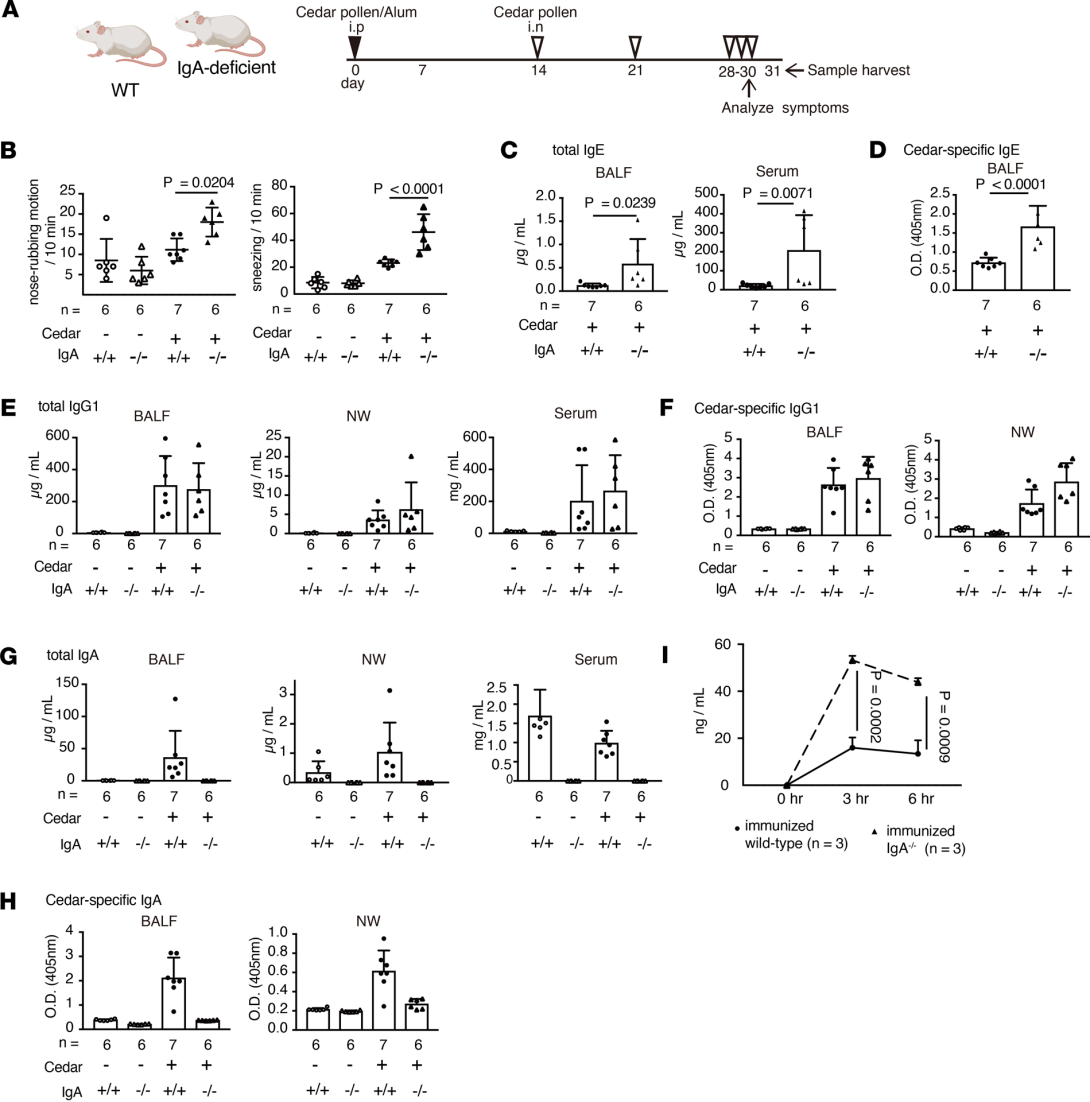
IgA is required for the regulation of the pathology in hay fever. (**A**) A schematic of the hay fever model in WT and IgA-deficient mice. (**B**) The rate of nose-rubbing motion and sneezing by administration of cedar pollen via the intranasal route (*n* = 6–7). (**C** and **D**) Total (**C**) and cedar-pollen-specific (**D**) IgE (*n* = 6–7). (**E** and **F**) Total (**E**) and cedar-pollen-specific (**F**) IgG1 (*n* = 6–7). (**G** and **H**) Total (**G**) and cedar-pollen-specific (**H**) IgA (*n* = 6–7). (**I**) The concentration of FITC-labeled cedar pollen in the serum (solid line: immunized WT mice; dashed line; immunized IgA^–/–^ mice) (*n* = 3). Statistical analysis was performed by 1-way ANOVA with Tukey’s multiple-comparison test (**B** and **E**–**H**) or unpaired, 2-tailed Student’s *t* test (**C**, **D**, and **I**). Data are expressed as mean ± SD in **B**–**I**.

To reveal the importance of IgA in hay fever, we analyzed the susceptibility of cedar-pollen-induced hay fever in IgA-deficient mice (Figure 1A). According to an assessment of rhinitis, pollen-inoculated IgA-deficient mice showed significantly increased levels of symptoms compared with pollen-inoculated wild-type (WT) animals (Figure 1B). The increased symptoms were associated with an increase in total and cedar-pollen-specific IgE production, but not IgG1, in the bronchoalveolar lavage fluid (BALF) and serum in IgA-deficient mice (Figure 1, C–F). (We could not detect IgE in the nasal wash [NW] because of the low titer of IgE in mice.) Next, we analyzed the production of Th2-related cytokines in cells derived from the mediastinal lymph nodes (medLNs) and nasal-associated lymphoid tissue (NALT). The absence of IgA was accompanied by significantly elevated levels of IL-4, IL-5, and IL-13 production (Supplemental Figure 1, A and B; supplemental material available online with this article; https://doi.org/10.1172/jci.insight.190240DS1). Infiltration of inflammatory immune cells such as lymphocytes, eosinophils, and neutrophils in the BALF and nasal passage (NP) was increased in IgA-deficient mice (Supplemental Figure 1, C and D). Because both the total IgA and cedar-pollen-specific IgA were increased in cedar pollen-inoculated WT mice compared with uninoculated mice (Figure 1, G and H), we hypothesized that secreted IgA blocks pollen invasion at the mucosal surface. To analyze whether IgA prevents the penetration of cedar pollen, we administered mice with fluorescein isothiocyanate–labeled (FITC-labeled) pollen via the intranasal route and evaluated the concentration of pollen in the serum. IgA-deficient mice showed higher levels of FITC-labeled cedar pollen in the serum (Figure 1I), indicating that mucosal IgA protects against cedar pollen penetration into the body.

### PKC activator induces IgA class switching while reducing IgE class switching in B cells.

**Figure 2:**
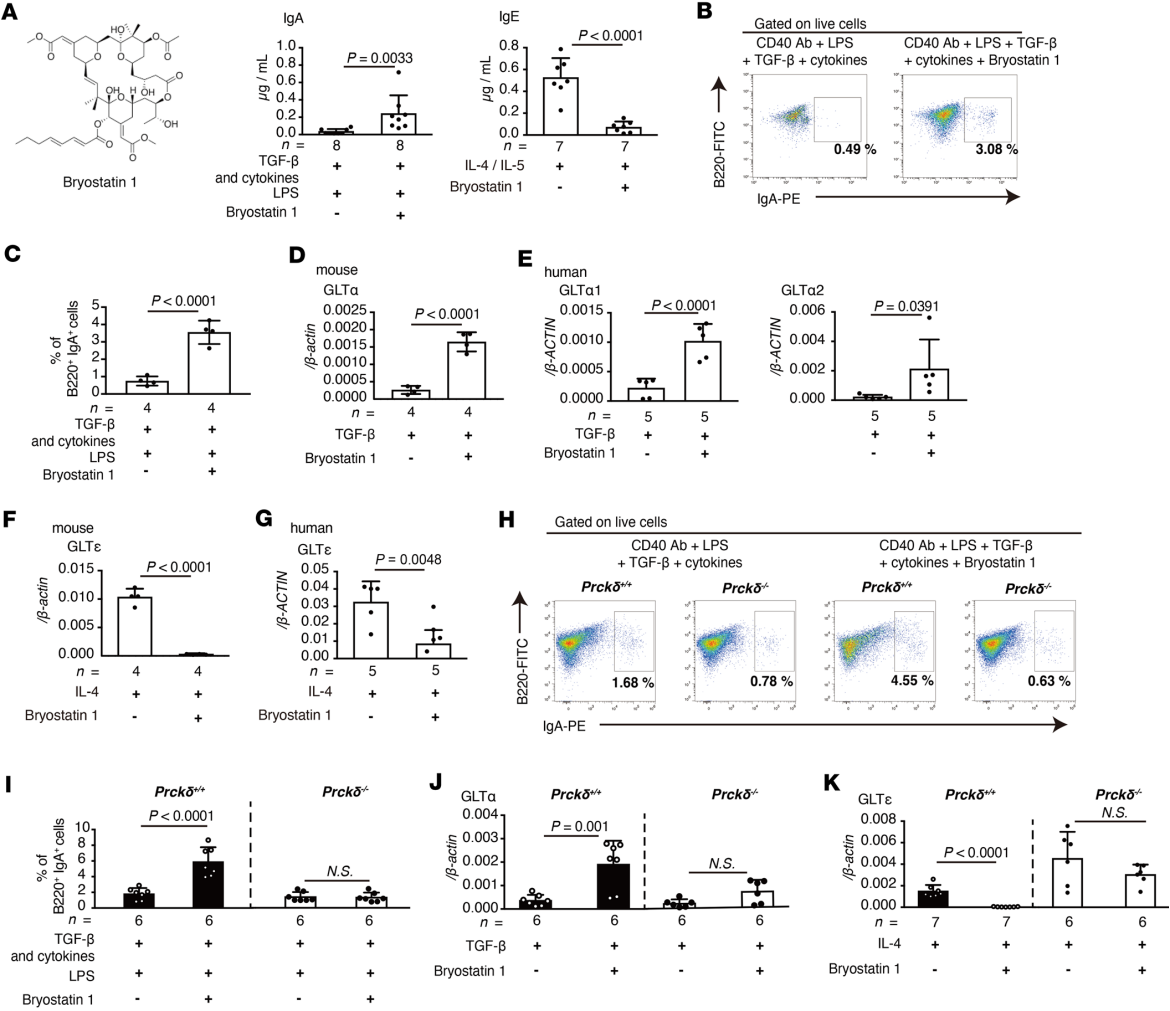
Bryostatin 1 enhances IgA class switching and suppresses IgE class switching through PKCδ activation. (**A**) Production of IgA and IgE in the supernatant of cultured WT B cells with indicated stimulation (left: structure of bryostatin 1). (**B** and **C**) Flow cytometry of IgA^+^ B cells. (**B**) Representative plots of cultured naive B cells with or without bryostatin 1 under stimulation with LPS and cytokines. (**C**) Frequency of IgA^+^B220^+^ cells (*n* = 4). (**D**) Expression of GLTα in cultured mouse B cells (*n* = 4). (**E**) Expression of GLTα1 and GLTα2 in cultured human B cells (*n* = 5). (**F**) Expression of GLTε in cultured mouse B cells measured (*n* = 4). (**G**) Expression of GLTε in cultured human B cells determined (*n* = 5). (**H** and **I**) Flow cytometry of IgA^+^ B cells. (**H**) Representative plots of cultured naive B cells with or without bryostatin 1 in WT and *Prckd*^–/–^ B cells under IgA class-switching-inducible condition. (**I**) Frequency of IgA^+^B220^+^ cells (*n* = 6). (**J**) Expression of GLTα in cultured mouse WT and *Prckd*^–/–^ B cells (*n* = 6). (**K**) Expression of GLTε in cultured mouse WT (*n* = 7) and *Prckd*^–/–^ (*n* = 6) B cells. Statistical analysis was performed by unpaired, 2-tailed Student’s *t* test (**A**, **C**–**G**, and **I**–**K**). Data are expressed as mean ± SD in **A**, **C**–**G**, and **I**–**K**.

Since IgA has a regulatory role in hay fever, we tried to identify the compound(s) that selectively induce IgA class switching in B cells based on the IgA production from spleen cells by ELISA. Of the chemical compound library consisting of 3,337 compounds, IgA production in the culture supernatant of splenocytes occurred for 22 compounds (threshold for IgA induction was set at OD_405_ ≥ 0.5) (Supplemental Figure 2A). In the next step, we measured IgA, IgE, and IgG production to identify IgA-specific inducible compound(s). Among them, 3 compounds selectively induced IgA but not IgE and IgG1 production: sapintoxin D, phorbol 12-myristate 13-acetate (PMA), and resiniferous 9,13,14-orthophenyl acetate (ROPA) (Supplemental Figure 2B). All 3 chemical compounds are protein kinase C (PKC) activators. We observed that the 3 compounds (all commercially available compounds) selectively induced IgA production but not IgE and IgG1 (Supplemental Figure 2C). Because PKC activators are known to be carcinogens (18, 19), we sought a noncarcinogenic PKC activator. Bryostatin 1 is reported as a noncarcinogenic PKC activator (20, 21), and clinical trials of bryostatin 1 for diseases including solid tumor, Alzheimer, and AIDS are already in progress (22–24). For these reasons, we focused on bryostatin 1 as a potential clinically acceptable PKC activator. Then, we confirmed that bryostatin 1 selectively induced IgA production while suppressing IgE production in B cells (Figure 2A).

Next, we analyzed whether bryostatin 1 affects IgA class switching. Compared with LPS and cytokine stimulation, including TGF-β, IL-4, IL-5, and B cell–activating factor (BAFF), the addition of bryostatin 1 to mouse naive B cells significantly induced IgA^+^ B cells (Figure 2, B and C), indicating that bryostatin 1 directly acts on B cells and enhances IgA class switching. To reveal the mechanism by which bryostatin 1 induces IgA^+^ B cells, we analyzed the expression of GLTα and AID in B cells. Although expression of AID was not affected in both mouse and human B cells by bryostatin 1 (Supplemental Figure 3, A–D), GLTα expression was significantly increased by the addition of bryostatin 1 in the TGF-β–stimulated condition in the mouse and human B cells (Figure 2, D and E). Class-switch recombination (CSR) causes the expression of circle transcript α (CTα) from excised circular DNA and post-switched transcript α (PSTα) from a recomposed genomic region in a heavy chain locus of immunoglobulin (3, 5). Bryostatin 1 also significantly enhanced the expression of these transcripts (Supplemental Figure 3E).

We next analyzed whether bryostatin 1 affects the class switching of other isotypes. Stimulation with IL-4 led to the expression of GLTε, PSTε, and CTε, but the addition of bryostatin 1 significantly suppressed the expression of these transcripts in mouse and human B cells (Figure 2, F and G, and Supplemental Figure 3F).

The efficiency of IgG1 class switching was not affected by bryostatin 1 (Supplemental Figure 3, G–I). In addition, GLTγ2b and GLTγ3 expression was not affected by bryostatin 1 (Supplemental Figure 3J). These results highlight that bryostatin 1 enhanced IgA class switching selectively, whereas it impaired IgE class switching dramatically in mouse and human B cells by regulating expression of GLTα and GLTε, respectively, but not affecting the other GLTs. Because previous research reported that bryostatin 1 activates Toll-like receptor 4 (TLR4), we analyzed whether TLR4 signaling is involved in bryostatin 1–mediated regulation of GLT expression (25). Bryostatin 1 induced an increase in GLTα and a reduction in GLTε in TLR4-deficient B cells (Supplemental Figure 4, A and B), indicating that TLR4 is not related to the regulation of GLTs in B cells.

We also analyzed whether bryostatin 1 affects the survival and proliferation of B cells. Bryostatin 1–treated B cells showed the same tendency of cell survival and proliferation (Supplemental Figure 5, A–C), indicating that bryostatin 1 did not affect B cell survival and proliferation.

### Bryostatin 1 acts on the PKCδ isozyme to regulate IgA and IgE class switching in B cells.

We next determined which PKC isozyme is activated by bryostatin 1 in B cells. We pretreated B cells with 12 PKC inhibitors, which have different inhibitory spectra for PKC isozyme(s), and analyzed the expression of GLTα and GLTε in the bryostatin 1–treated condition. PKCδ inhibitors abolished bryostatin 1–mediated regulation of GLTα and GLTε expression (Supplemental Figure 6, A and B). In correlation with inhibitor analysis, among PKC isozymes, PKCδ was highly expressed in human and mouse B cells (Supplemental Figure 6, C and D). In addition, IgA^+^ B cells were not induced in PKCδ-deficient B cells (Figure 2, H and I), and the level of GLT expression in PKCδ-deficient B cells was not changed by bryostatin 1 (Figure 2J). Correspondingly, the PSTα and CTα expression and IgA production in PKCδ-deficient B cells were not affected by bryostatin 1 (Supplemental Figure 7, A and B).

We next analyzed whether bryostatin 1 suppresses IgE class switching in PKCδ-deficient B cells. As expected, bryostatin 1 attenuated the GLTε, PSTε, and CTε expression and production of IgE induced by IL-4 in WT B cells but not in PKCδ-deficient cells (Figure 2K and Supplemental Figure 7, C and D). Taken together, these results show that bryostatin 1 acts on PKCδ to enhance IgA class switching and attenuate IgE class switching in B cells.

### Bryostatin 1/PKCδ/ERK axis activates RUNX1 through phosphorylation and enhances GLTα expression.

**Figure 3:**
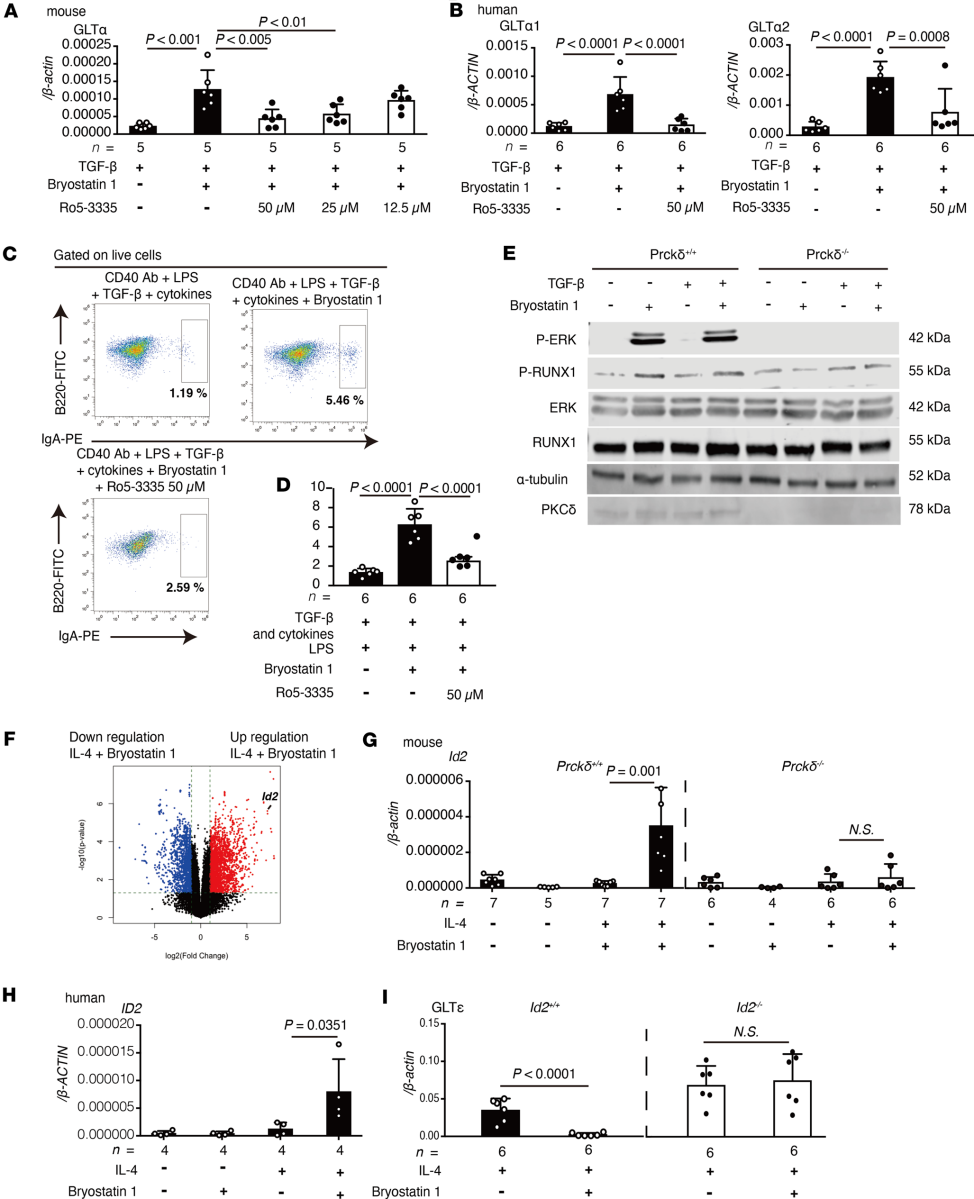
Bryostatin 1 induces GLTα expression through RUNX1 activation and suppresses GLTε expression through ID2 expression. (**A**) Expression of GLTα in cultured mouse B cells with or without Ro5-3335 at indicated concentrations (*n* = 5). (**B**) Expression of GLTα1 and GLTα2 in cultured human B cells with or without Ro5-3335 (*n* = 6). (**C** and **D**) Flow cytometry of IgA^+^ B cells. (**C**) Representative plots of cultured naive B cells with or without Ro5-3335 under IgA class-switching-inducible condition. (**D**) Frequency of IgA^+^B220^+^ cells (*n* = 6). (**E**) Immunoblot analysis of p-ERK, p-RUNX1, ERK, RUNX1, α-tubulin, and PKCδ in B cells from WT or *Prckd*^–/–^ mice. (**F**) Volcano plot of differentially expressed genes in bulk RNA sequencing of IL-4–stimulated B cells cultured with or without bryostatin 1. (**G**) Expression of *Id2* in cultured mouse WT (*n* = 5–7) and *Prckd*^–/–^ (*n* = 4–6) B cells with or without bryostatin 1. (**H**) Expression of *ID2* in cultured human B cells with or without bryostatin 1 (*n* = 4). (**I**) Expression of GLTε in cultured WT and *Id2*^–/–^ (*n* = 6) B cells. Statistical analysis was performed by 1-way ANOVA with Tukey’s multiple-comparison test (**A**, **B**, **D**, **G**, and **H**) and unpaired, 2-tailed Student’s *t* test (**I**). Data are expressed as mean ± SD in **A**, **B**, **D**, and **G**–**I**.

Because it has been reported that PKCδ activates the MAPK/ERK pathway, we examined whether its signaling is related to the bryostatin 1–induced GLTα expression (25–27). Inhibition of MEK signaling by U0126 was shown to suppress bryostatin 1–mediated GLT expression in a dose-dependent manner (Supplemental Figure 8A). Since the MEK/ERK pathway is known to activate diverse downstream transcription factors (28, 29), we treated B cells with inhibitors of them and evaluated the GLTα expression in B cells. Among 13 inhibitors, Ro5-3335, a selective inhibitor of RUNX1, interfered with bryostatin 1–induced upregulation of GLTα in mouse and human B cells (Supplemental Figure 8B and Figure 3, A and B). We confirmed that RUNX1 was highly expressed in mouse and human B cells (Supplemental Figure 8, C and D). Induction of IgA^+^ B cells, PSTα and CTα expression, and production of IgA were significantly reduced by treatment with Ro5-3335 (Figure 3, C and D, and Supplemental Figure 8, E and F). Because it has been reported that the MEK/ERK pathway regulates the phosphorylation of the RUNX family of proteins, we next analyzed whether bryostatin 1 phosphorylates ERK and RUNX1 in B cells (30–32). Bryostatin 1 induced phosphorylation of ERK and RUNX1 in WT B cells but not in PKCδ-deficient cells (Figure 3E and Supplemental Figure 9, A and B). These findings suggest that bryostatin 1 activates the PKCδ/MEK/ERK/RUNX 1 pathway, resulting in the enhancement of IgA class switching in B cells through induction of GLTα expression.

### ID2 plays a pivotal role in the bryostatin 1–mediated downregulation of IgE class switching.

We performed mRNA-sequencing analysis to elucidate the inhibitory mechanism of IgE class switching by bryostatin 1. Stimulation of B cells with bryostatin 1 and IL-4 or TGF-β induced a dramatic change in gene expression (Figure 3F and Supplemental Figure 10A). Among them, the expression level of inhibitor of DNA binding 2 (ID2) was significantly increased by bryostatin 1 (Figure 3F). We focused on ID2 because it is already reported as a suppressor of GLTε expression (33, 34). We revealed that ID2 expression is induced by IL-4 and bryostatin 1 stimulation in WT mouse and human B cells but not in PKCδ-deficient cells (Figure 3, G and H). Bryostatin 1–mediated ID2 induction was also observed at the protein level (Supplemental Figure 11, A and B). As expected, bryostatin 1–mediated suppression of GLTε expression did not appear in ID2-deficient B cells (Figure 3I and Supplemental Figure 12, A and B). These findings support the idea that bryostatin 1–induced ID2 expression through PKC signaling attenuated IgE class switching by reduction of GLTε expression.

**Figure 4:**
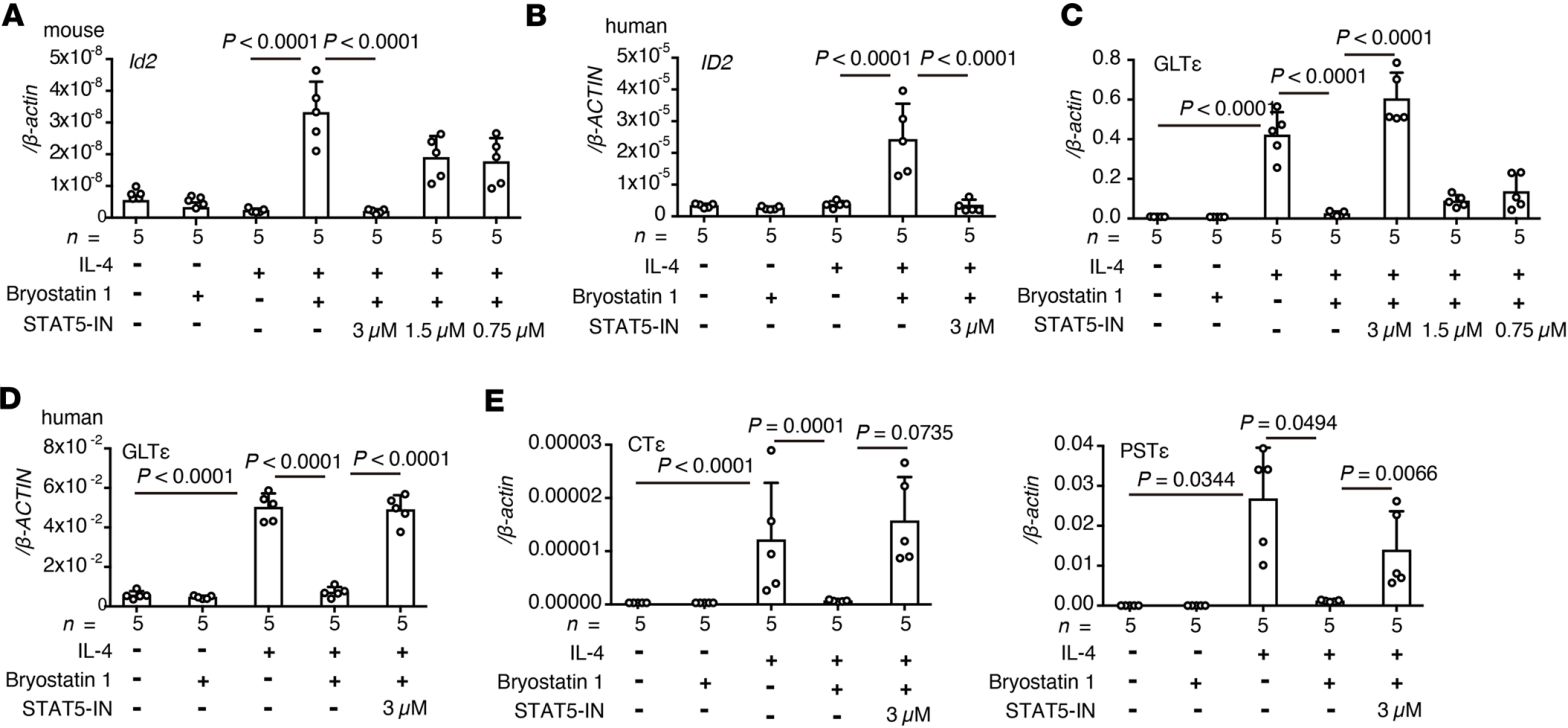
STAT5 is involved in bryostatin 1–mediated suppression of GLTε expression through ID2 induction. (**A** and **B**) Expression of ID2 in cultured mouse and human B cells with indicated stimulation. Cells were pretreated with STAT5 inhibitor (STAT5-IN) before stimulation with bryostatin 1 (**A**: mouse; **B**: human) (*n* = 5). (**C** and **D**) Expression of GLTε in cultured mouse and human B cells with indicated stimulation. Cells were pretreated with STAT5-IN before stimulation with bryostatin 1 (**C**: mouse; **D**: human) (*n* = 5). (**E**) Expression of PSTε and CTε in cultured mouse B cells with indicated stimulation. Cells were pretreated with STAT5-IN before stimulation with bryostatin 1 (*n* = 5). Statistical analysis was performed by 1-way ANOVA with Tukey’s multiple-comparison test (**A**–**E**). Data are expressed as mean ± SD in **A**–**E**.

We next analyzed the transcription factor involved in the expression of ID2. It has been reported that ID2 expression is regulated by STAT5 (35, 36), and the activation of the STAT family is controlled by phosphorylation through PKC (37, 38). Therefore, we analyzed whether bryostatin 1 regulates ID2 expression through STAT5 signaling. Induction of ID2 expression was attenuated by treating mouse and human B cells with a STAT5 inhibitor (Figure 4, A and B). STAT5 inhibition interfered with bryostatin 1–mediated suppression of GLTε, CTε, and PSTε expression in mouse and human B cells (Figure 4, C–E). Thus, bryostatin 1 induces ID2 expression via the PKCδ/STAT5 pathway to suppress the expression of GLTε.

### Bryostatin 1 attenuated allergic responses in mouse models of cedar-pollen-mediated hay fever.

**Figure 5:**
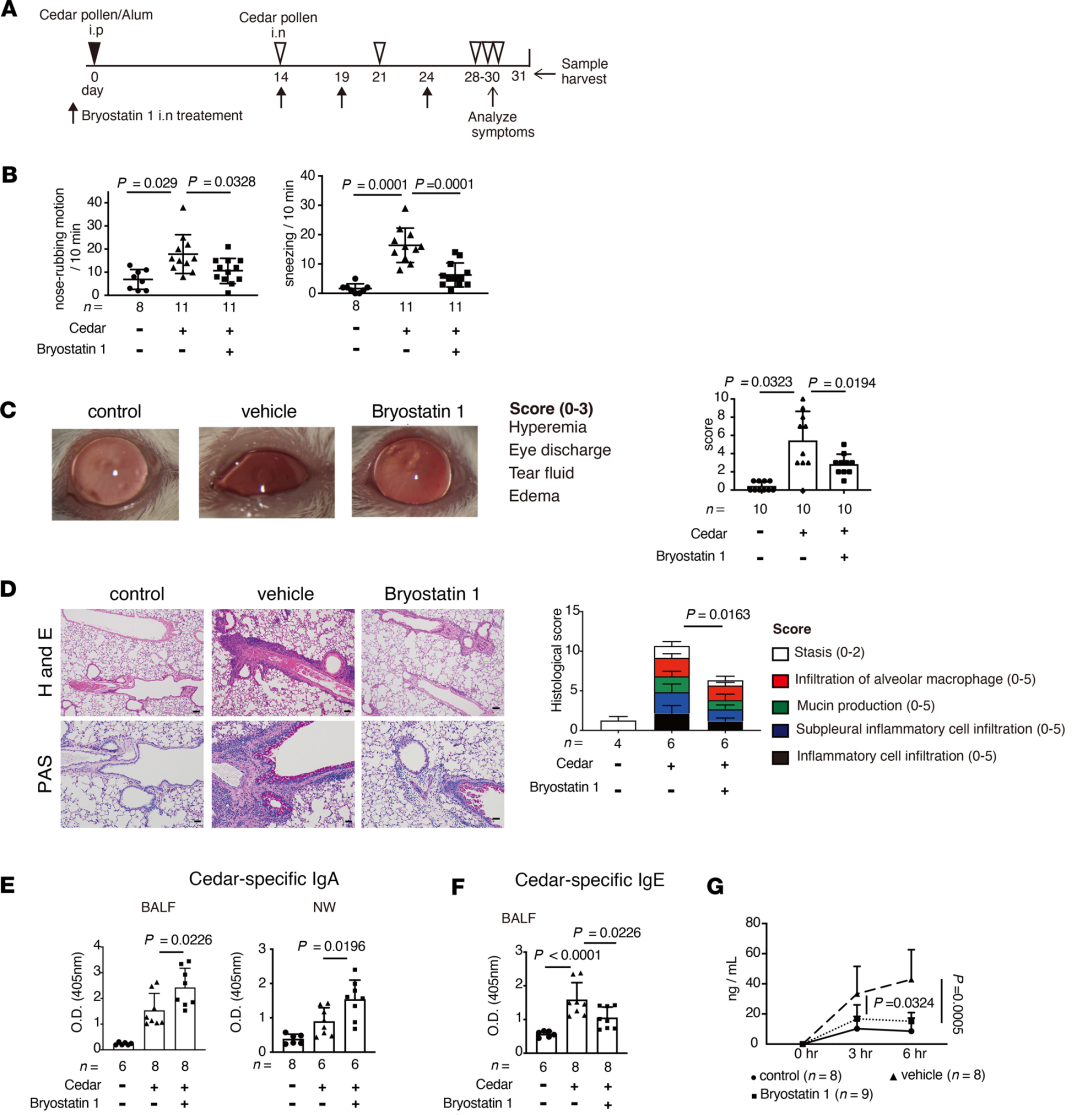
Bryostatin 1 ameliorates cedar-pollen-induced hay fever. (**A**) A schematic of cedar-pollen-induced hay fever model mice with or without bryostatin 1 treatment. (**B**) The rate of nose-rubbing motion and sneezing mediated by administration of cedar pollen via the intranasal route (*n* = 8–13). (**C**) Representative eye images and clinical sore of conjunctivitis (*n* = 10). (**D**) Representative lung histological images of H&E staining and PAS staining. The clinical score was evaluated by the indicated parameter (*n* = 4–6). (**E**) Cedar-pollen-specific IgA in the BALF and NW (*n* = 8–12). (**F**). Cedar-pollen-specific IgE in the BALF (*n* = 8–12). (**G**) The concentration of FITC-labeled cedar pollen in the serum (solid line: control mice; dashed line; immunized vehicle-treated mice; broken line; immunized bryostatin 1–treated mice) (*n =* 8–9). Statistical analysis was performed by 1-way ANOVA with Tukey’s multiple-comparison test (**B**–**G**). Data are expressed as mean ± SD in **B**–**G**.

To clarify whether bryostatin 1 can ameliorate allergic responses, cedar-pollen-induced hay fever model mice were administered bryostatin 1 via the intranasal route (Figure 5A). Subsequent cedar pollen administration via the intranasal route in inoculated mice induced hay fever symptoms, including rhinitis, conjunctivitis, and lung inflammation, but these symptoms were significantly reduced compared with untreated animals (Figure 5, B–D). Bryostatin 1 administration enhanced the production of total and pollen-specific IgA in the BALF and NW (Figure 5E and Supplemental Figure 13A). In contrast, IgE production was reduced in the BALF and serum by bryostatin 1 (Figure 5F and Supplemental Figure 13B). According to in vitro experiments, IgG1 production was virtually unaffected by treatment with bryostatin 1 (Supplemental Figure 13C).

Next, we analyzed whether bryostatin 1–induced IgA could prevent pollen invasion. In correlation with the results from IgA-deficient mice (Figure 1I), bryostatin 1–treated mice showed significantly lower levels of FITC-labeled cedar pollen in the serum (Figure 5G), suggesting that enhanced IgA production in the mucosa by bryostatin 1 prevents pollen penetration. In addition, the production of Th2-related cytokines from medLN and NALT cells and immune cell infiltration in the BALF and NP were reduced in bryostatin 1–treated mice (Supplemental Figure 13, D–G). Moreover, treatment with bryostatin 1 significantly enhanced the induction of IgA^+^ B cells in the medLNs and NALT (Supplemental Figure 13, H and I, and Supplemental Figure 14A). In contrast, the germinal center reaction was nearly identical between bryostatin 1–treated and untreated mice (Supplemental Figure 14B). Thus, the enhancement of IgA class switching by bryostatin 1 in vivo alleviates hay fever in mouse models.

### Bryostatin 1 regulates antibody production and attenuates the pathogenesis of hay fever in a PKCδ/RUNX1- and STAT5-signaling-dependent manner.

**Figure 6:**
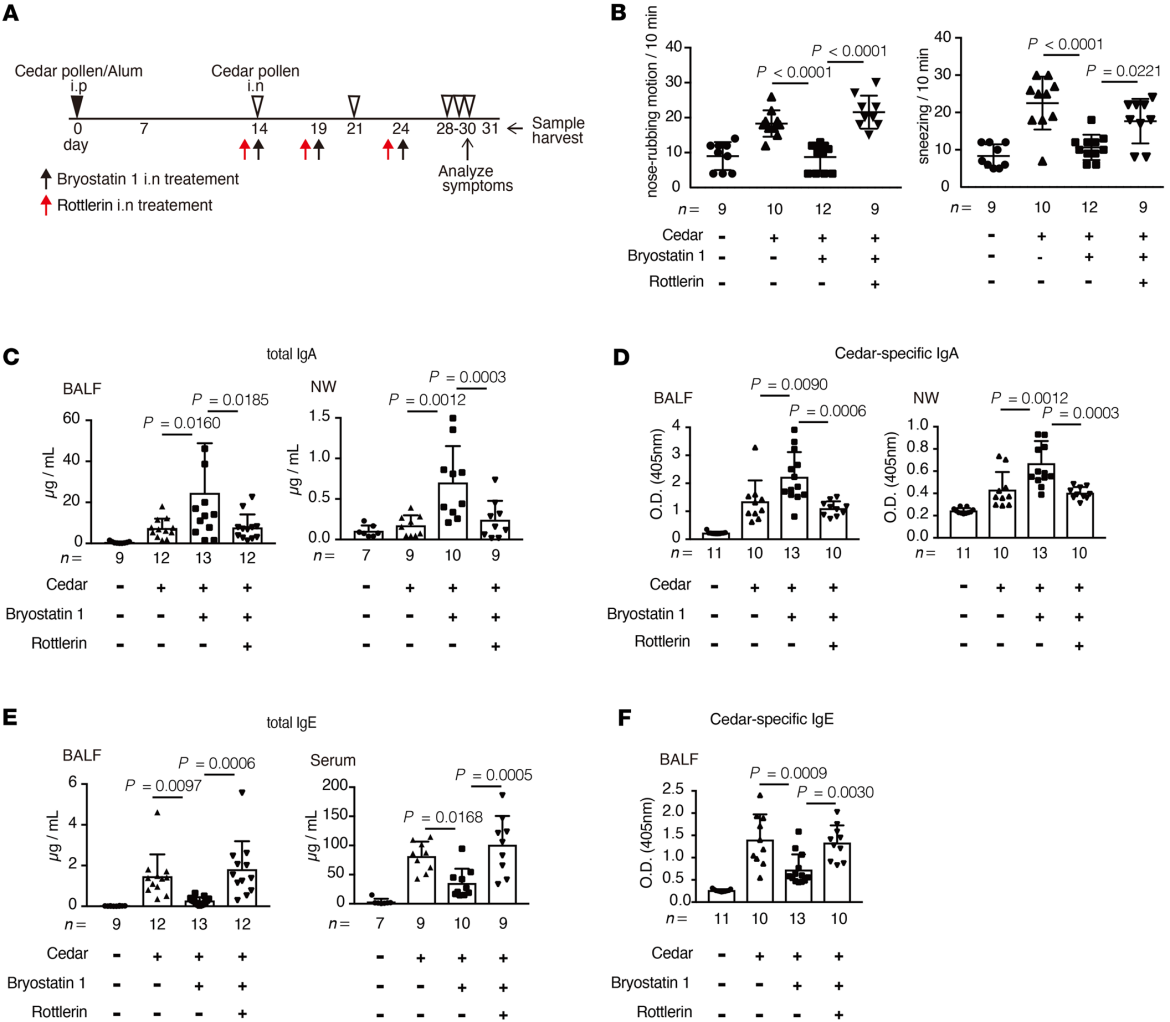
Bryostatin 1 alleviates cedar-pollen-induced hay fever in a PKCδ-dependent manner. (**A**) A schematic of the study in cedar-pollen-induced hay fever model mice with or without treatment with PKCδ inhibitor. (**B**) The rate of nose-rubbing motion and sneezing mediated by the administration of cedar pollen via the intranasal route (*n* = 9–12). (**C** and **D**) Total (**C**) and cedar-pollen-specific (**D**) IgA in the BALF and NW (*n* = 7–13). (**E** and **F**) Total (**E**) and cedar-pollen-specific (**F**) IgE in the BALF and serum (*n* = 7–13). Statistical analysis was performed by 1-way ANOVA with Tukey’s multiple-comparison test (**B**–**F**). Data are expressed as mean ± SD in **B**–**F**.

To analyze the dependency on PKCδ, we next administered mice with the PKCδ-specific inhibitor rottlerin before administration of bryostatin 1 via the intranasal route (Figure 6A) (27, 39, 40). We found that treatment with bryostatin 1 alongside rottlerin failed to alleviate hay fever–associated symptoms (Figure 6B). Furthermore, bryostatin 1–mediated enhancement of total and cedar-pollen-specific IgA and suppression of total and cedar-pollen-specific IgE production in the BALF, NW, and serum were diminished by the administration of rottlerin (Figure 6, C–F). The effect of bryostatin 1 on the production of Th2-related cytokines from medLN and NALT cells was also absent in mice treated with rottlerin (Supplemental Figure 15, A and B). Treatment with rottlerin inhibited the bryostatin 1–mediated reduction in immune cell infiltration in the BALF and NP (Supplemental Figure 15, C and D). These results demonstrate that the bryostatin 1/PKCδ axis ameliorated hay fever by regulating IgA and IgE class switching.

To address the question whether bryostatin 1 regulates the IgA and IgE production through RUNX1, STAT5, and ID2 in disease conditions, we administered the hay fever model mice with a RUNX1 or STAT5 inhibitor, respectively, via the intranasal route before treatment with bryostatin 1 (Supplemental Figure 16A and Supplemental Figure 17A). The administration of the RUNX1 inhibitor reversed the enhancement of IgA production but not the reduction in IgE production associated with the abolishment of symptom improvement by bryostatin 1 (Supplemental Figure 16, B–F). In contrast, the administration of the STAT5 inhibitor reversed the downregulation of IgE production but not the enhancement of IgA production mediated by bryostatin 1, with abolishment of symptom improvement by bryostatin 1 (Supplemental Figure 17, B–F). In correlation with STAT5 inhibition, treatment with bryostatin 1 in ID2-deficient mice failed to alleviate hay fever–associated symptoms Supplemental Figure 18A). Furthermore, the bryostatin 1–mediated reduction in total and cedar-pollen-specific IgE, but not enhancement of IgA production, was diminished in ID2-deficient mice (Supplemental Figure 18, B–D). These results emphasize that both enhanced IgA and reduced IgE production in the mucosal sites are necessary to improve fay fever conditions with bryostatin 1 treatment.

**Figure 7:**
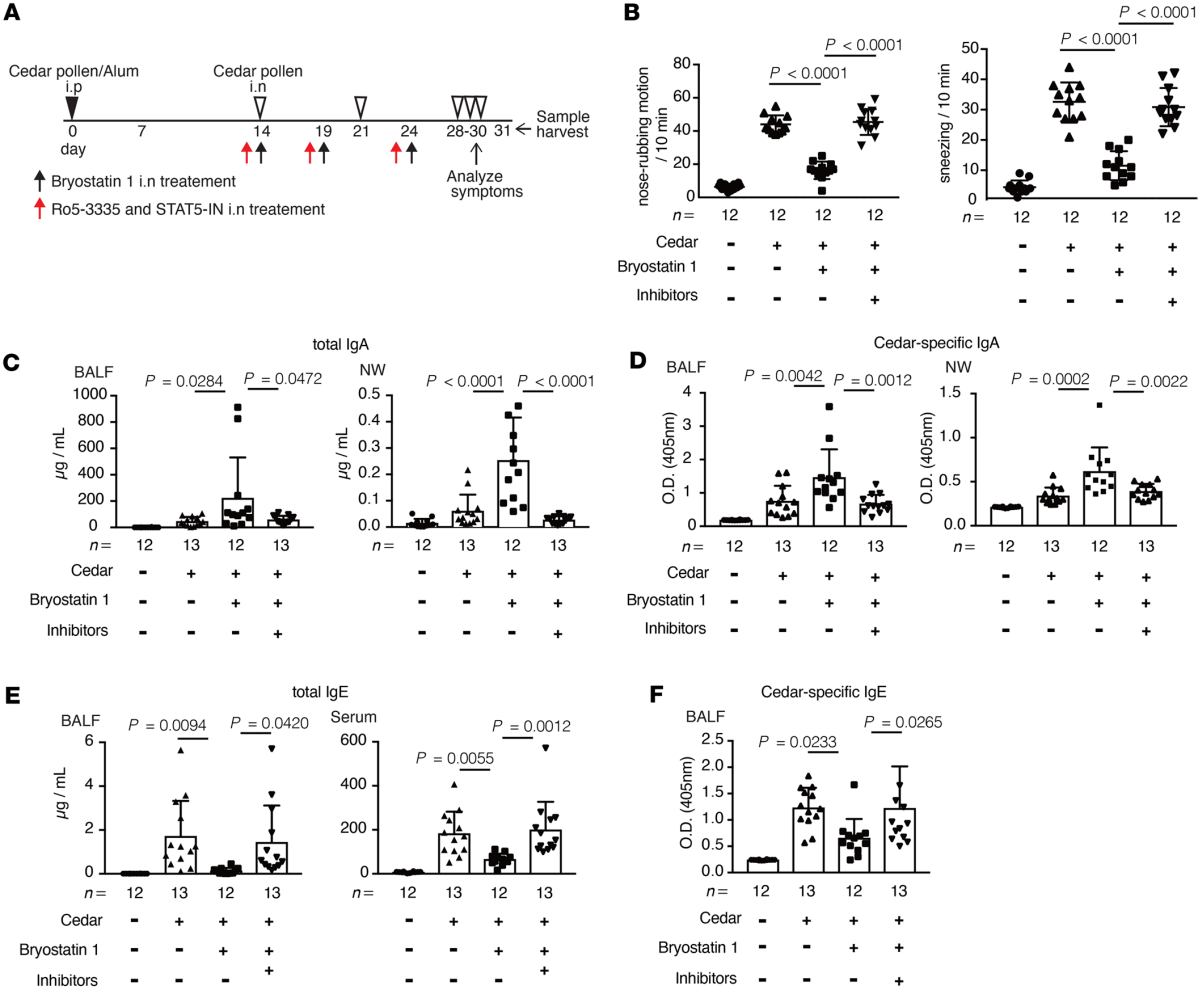
Bryostatin 1 alleviates cedar-pollen-induced hay fever in a RUNX1- and STAT5-dependent manner. (**A**) A schematic of the study in cedar-pollen-induced hay fever model mice with or without treatment with RUNX1 and STAT5 inhibitor. (**B**) The rate of nose-rubbing motion and sneezing mediated by the administration of cedar pollen via the intranasal route (*n* = 12). (**C** and **D**) Total (**C**) and cedar-pollen-specific (**D**) IgA in the BALF and NW (*n* = 12–13). (**E** and **F**) Total (**E**) and cedar-pollen-specific (**F**) IgE in the BALF and serum (*n* = 12–13). Statistical analysis was performed by 1-way ANOVA with Tukey’s multiple-comparison test (**B**–**F**). Data are expressed as mean ± SD in **B**–**F**.

To analyze whether inhibition of both RUNX1 and STAT5 annuls the effects of bryostatin 1, we administered hay fever model mice with both inhibitors via the intranasal route before the treatment with bryostatin 1 and assessed the severity of the disease (Figure 7A). We found that pretreatment with RUNX1 and STAT5 inhibitors attenuated the therapeutic effects of bryostatin 1 (Figure 7B). In addition, bryostatin 1–mediated phenomena, including enhancement of IgA production, suppression of IgE and Th2-related cytokine production, and reduction in immune cell infiltration were absent in mice treated with both inhibitors (Figure 7, C–F, and Supplemental Figure 19, A–D). These findings provide further evidence that bryostatin 1 ameliorates hay fever in a PKCδ/RUNX1- and STAT5-signaling-dependent manner, as suggested by our in vitro experiments.

### Bryostatin 1 acts as a curative treatment for hay fever.

**Figure 8:**
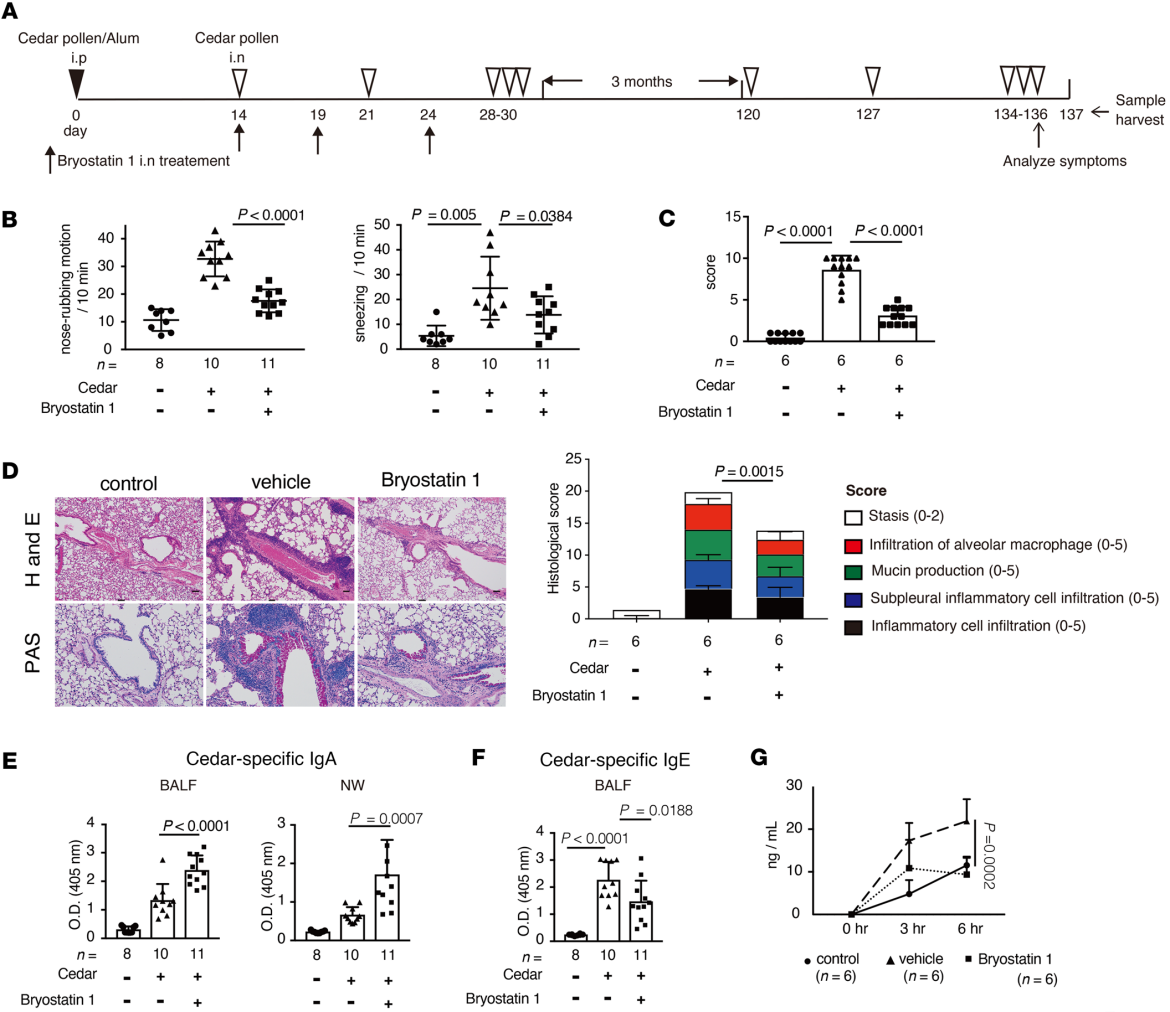
Bryostatin 1 ameliorates cedar-pollen-induced seasonal exposure hay fever model. (**A**) A schematic of cedar-pollen-induced hay fever model mice with or without bryostatin 1 treatment (**B**) The rate of nose-rubbing motion and sneezing mediated by administration of cedar pollen via the intranasal route (*n* = 8–11). (**C**) Clinical sore of conjunctivitis (*n* = 6). (**D**) Representative lung histological images of H&E staining and PAS staining. The clinical score was evaluated by the indicated parameter (*n* = 6). (**E**) Cedar-pollen-specific IgA (*n* = 8–11). (**F**) Cedar-pollen-specific IgE (*n* = 8–11). (**G**) The concentration of FITC-labeled cedar pollen in the serum (solid line: control mice; dashed line; immunized vehicle-treated mice; broken line; immunized bryostatin 1–treated mice) (*n =* 6). Statistical analysis was performed by 1-way ANOVA with Tukey’s multiple-comparison test (**B**–**G**). Data are expressed as mean ± SD in **B**–**G**.

Because it remains unclear whether bryostatin 1 works as a symptomatic or curative treatment in the previous protocol (Figure 5A), we established a protocol to evaluate the therapeutic outcome of bryostatin 1. We inoculated mice with cedar pollen followed by intranasal administration of bryostatin 1, as in the previous protocol. Three months later, we administered cedar pollen to the mice again to mimic seasonal exposure. To evaluate the curative effect of bryostatin 1, we did not re-treat mice with bryostatin 1 when cedar pollen was administered to animals following the 3-month interlude (Figure 8A). Mice that had been treated 3 months previously with bryostatin 1 still showed reduced levels of hay fever–associated symptoms compared with untreated animals (Figure 8, B–D). Treatment with bryostatin 1 suppressed allergy-related responses, including IgE and Th2-related cytokine production and immune cell infiltration, which was associated with enhancement of IgA production and prevention of cedar pollen penetration in the mucosa (Figure 8, E–G, and Supplemental Figure 20, A–F). These results suggest that bryostatin 1 provides sustained beneficial effects against pollen-induced hay fever. Therefore, bryostatin 1 is a promising candidate for the curative treatment of hay fever.

## Discussion

We demonstrated that the increase in allergen invasion into the body is associated with the symptoms of hay fever in IgA-deficient mice. In association with increasing pollen penetration, IgE and Th2-related cytokine production was also enhanced in these mice. These results raise the possibility that secreted IgA prevents the penetration of allergens in the mucosal sites. We have shown that activation of PKCδ regulates IgA and IgE class switching in B cells and confers protection against hay fever–associated allergic responses through induction of IgA and a reduction in IgE production.

PKC is ubiquitously expressed and known to be activated by a variety of stimuli. Its downstream signals are also diverse. In this study, we show for the first time to our knowledge that bryostatin 1, a noncarcinogenic PKC activator, can suppress pollen allergy by 2 identified downstream pathways, thereby shifting the direction of B cell class switching from IgE to IgA by regulating GLT expression.

Previous reports have already revealed that PKCδ plays a pivotal role in the regulation of immune homeostasis by showing that PKCδ-deficient mice showed dysfunction in a number of immune cells, including T cells, B cells, and phagocytes (41–44). In association with immune cell disorder, PKCδ-deficient mice develop systemic autoimmune disorder with severe humoral autoimmunity by loss of tolerance in peripheral B cell development (45). Also, patients with genetic abnormalities of PKCδ have been reported to represent humoral autoimmune diseases, particularly symptoms associated with systemic lupus erythematosus (46). Therefore, control of PKCδ signaling by its activator can be a promising target for immune disorder–related diseases, including allergic diseases.

The current study contributes further evidence of the therapeutic benefits of controlling PKCδ signaling, in particular for alleviating hay fever, which is also an immune disorder–related disease. Furthermore, our study highlights the efficacy of bryostatin 1 in controlling PKCδ signaling in B cells. Among the multiple signaling pathways downstream of PKCδ, we believe that the pathway we have identified here is a critical one because it does not simply strengthen the whole antibody response, but is important for maintaining a delicate immune balance by simultaneously triggering an isotype-specific increase or decrease in transcription. In addition to mouse studies, human B cells also showed the same regulation of IgA and IgE GLT regulation mediated by bryostatin 1. These findings allow us to speculate that bryostatin 1 also alleviates hay fever symptoms in humans by blocking the penetration of pollen antigens.

Naive B cells require the expression of GLTα and AID to induce IgA class switching. TGF-β is well known as an essential cytokine for IgA class switching via the expression of GLTα through the transcription factors SMAD2 and SMAD3. Bryostatin 1 could not induce GLT expression without TGF-β (data not shown). In the presence of TGF-β, we demonstrated that phosphorylation of RUNX1 mediated by bryostatin 1 enhanced the expression of GLTα through activation of the PKCδ/ERK signaling pathway. In addition, RUNX1 was dominantly expressed and there are RUNX1-binding sequences in the promoter region of GLTα in both mouse and human B cells. Further studies are required to determine the interaction between SMAD2, SMAD3, and RUNX1 for GLTα expression.

Previous studies demonstrated that bryostatin 1 acts on T cells (47) and macrophages (48). While another report has demonstrated that bryostatin 1 induces the production of Th2-related cytokines (49), in our study, bryostatin 1 suppressed Th2-related cytokines in an antigen-dependent manner. We speculate that, by blocking pollen invasion mediated by secreted IgA, antigen-specific T cell stimulation itself was not induced even in the presence of bryostatin 1 in vivo. Thus, we believe it is a novel strategy for the treatment of hay fever compared with current treatments such as histamine blockers and anti-IgE, anti–IL-4, and anti–IL-13 antibodies. In other words, bryostatin 1 does not block but shifts the immune responses from IgE toward IgA. This raises the possibility that bryostatin 1 may modulate not only allergic diseases but also other immune abnormalities such as an autoimmune disorder by changing the direction of immune responses. Further studies are necessary to investigate whether bryostatin 1 improves other immune abnormalities, how it acts on individual immune cells, and how it normalizes the overall immune status.

## Methods

### Sex as a biological variable.

Our study including in vitro and in vivo experiments examined male and female animals, and similar findings are reported for both sexes.

### Mice.

BALB/c, C57BL/6J, and 129^+Ter^/SvJcl mice were purchased from Japan CLEA. IgA-deficient, PKCδ-deficient, and ID2-deficient mice (129^+Ter^/SvJcl background) were provided in-house. These mice were bred and maintained under specific pathogen–free (SPF) conditions at the animal facility of the Institute for Quantitative Bioscience, the University of Tokyo.

### Reagents.

Reagents are listed in Table 1.

### Cedar pollen extract–induced hay fever model.

On day 0, mice received 150 μg of cedar pollen (Cosmo Bio) and 25 mg of aluminum potassium sulfate (Nacalai) intraperitoneally in 200 μL phosphate-buffered saline (PBS). On days 14, 21, and 28–30, mice received 25 μg of cedar pollen via the intranasal route. The mice were administered with 20 ng bryostatin 1 via the intranasal route on days 14, 19, and 24. On day 30, symptoms of hay fever were observed. Sneezing and nasal rubbing were assessed for 10 minutes after intranasal administration of cedar pollen (50 μg in 40 μL PBS).

### Sample collection and processing from hay fever model mouse.

On day 31, mice were euthanized, and samples were collected. Noses were fragmented by scissors, and NPs were scraped with a spatula. The homogenized cell suspensions were sieved through 40 μm cell strainers (Falcon), and red blood cells were lysed. Single-cell suspensions of medLNs and NALT were obtained by homogenizing the organs. Cells were cultured and restimulated with or without pollen in RPMI 1640 medium containing 10 % FBS, 500 μg/mL penicillin, and 50 μM 2-mercaptoethanol. After 5 days, culture supernatants were collected for ELISA. In the case of inhibitor treatment, 5 μg rottlerin, 5 μg Ro5-3335, and 5 μg STAT5 inhibitor were administered to the mice via the intranasal route 1 hour before treatment with bryostatin 1.

### Analysis of conjunctivitis.

Clinical features were graded according to a scoring system. Each eye was assessed for hyperemia, eye discharge, tear fluid, and edema. Each parameter was graded on a scale of 0 to 3.

### Histology of the lung tissue.

Sections of the lung of uninoculated mice or cedar-pollen-inoculated mice were stained with hematoxylin and eosin (H&E) and periodic acid–Schiff (PAS). The lung sections were evaluated in a double-blind fashion.

### ELISA.

Antibody titer was measured by sandwich ELISA. Capture antibody or pollen was suspended in 0.05 M Na_2_CO_3_ and coated on ELISA plates. Capture antibodies were goat anti-mouse IgA (Southern Biotech), anti-mouse IgE (Southern Biotech), and IgG1 (Southern Biotech). After blocking with 1% bovine serum albumin (Wako) in PBS, the concentration of antibody was detected with alkaline phosphatase–conjugated (AP-conjugated) anti-mouse IgA (Southern Biotech), IgE (Southern Biotech), and IgG1 (Southern Biotech) with 1% bovine serum albumin in PBS and AP substrate (Sigma-Aldrich) in detection buffer (6.5 mM Na_2_CO_3_ and 18.5 mM NaHCO_3_). In detecting cedar-pollen-specific IgE, biotinylated anti-mouse IgE (Southern Biotech) and Streptavidin Alkaline Phosphatase (Southern Biotech) were utilized. After the development of the colorimetric reaction, the optical density (OD) values at 405 nm were measured by Tristar^2^ LB 942 (Berthold Technologies). IL-4, IL-5, and IL-13 concentrations in the collected samples were measured by Mouse IL-4, IL-5, and IL-13 ELISA Kit (Proteintech) according to the manufacturer’s protocol.

### FACS.

Harvested cells from BALF and NP were incubated with mouse anti-CD16/anti-CD32 antibody (BioLegend, 101301, clone 93) for 20 minutes at 4°C and stained with fluorescently conjugated antibody cocktails, including PerCP.Cy5.5 anti-mouse CD45 (BioLegend, 103131, clone 30-F11), PE anti-mouse Siglec-F (BioLegend, 155505, clone S17007L), Pacific Blue anti-mouse CD11c (BioLegend, 117321, clone N418), FITC anti-mouse CD3 (BioLegend, 100305, clone 145-2C11), FITC anti-mouse B220 (BioLegend, 103205, clone RA3-6B2), and PECy7 anti-mouse Gr-1 (BioLegend, 108415, clone RB6-8C5) in FACS buffer (1% FCS and 1 μM EDTA) in PBS for 20 minutes at 4°C. Dead cells were excluded with propidium iodide staining (Nacalai). Cells were analyzed with a Spectral Cell Analyzer SA3800 (SONY). The following antibodies were used for FACS staining: PerCP.Cy5.5 anti-mouse CD45 (BioLegend, 103131, clone 30-F11), anti-mouse Siglec-F (BioLegend, 155505, clone S17007L), Pacific Blue anti-mouse CD11c (BioLegend, 117321, clone N418), FITC anti-mouse CD3 (BioLegend, 100305, clone 145-2C11), FITC anti-mouse B220 (BioLegend, 103205, clone RA3-6B2), PECy7 anti-mouse Gr-1 (BioLegend, 108415, clone RB6-8C5), and PE anti-mouse IgA (Southern Biotech, 1040-09, polyclonal).

### Measurement of the permeability of cedar pollen.

Cedar pollen was labeled using a Pierce FITC Antibody Labeling Kit following the manufacturer’s protocol (Thermo Fisher Scientific). Briefly, 1 mg of cedar pollen in 0.5 mL of FITC labeling buffer was reacted for conjugation of FITC to protein and FITC-labeled cedar pollen was purified by Purification Resin column. Cedar-pollen-immunized mice received FITC-labeled cedar pollen in 30 μL PBS on day 30 via the intranasal route, and serum was collected 3 hours and 6 hours later. The fluorescence intensity was measured with a Tristar^2^ LB 942.

### Screening of IgA-inducible compounds.

In the first screening, mouse spleen cells (2 × 10^5^ cells) were cultured with individual compounds from the chemical compound library (3,337 compounds) provided by the Drug Discovery Initiative, The University of Tokyo, at 3.75 μM concentration in RPMI 1640 medium containing 500 ng/mL anti-mouse CD40 antibody (BioLegend), 10 % FBS, 500 μg/mL penicillin, and 50 μM 2-mercaptoethanol in a 96-well plate (200 μL/well). After 10 days of culture, the culture supernatant was collected and measured by ELISA to detect IgA antibody levels. Compounds with an OD_405_ of greater than 0.6 were determined as an IgA-inducible compounds. In the second screening, the production of IgA, IgE, and IgG1 by anti-CD40 antibody–stimulated splenocytes with 22 candidate compounds was measured. Among them, because of their IgA selective induction, sapintoxin D, PMA, and ROPA were analyzed by ELISA as the third screening. Whole splenocytes were stimulated by anti-mouse CD40 antibody with 0.8 nM bryostatin 1 or 20 μg/mL LPS alone. Seven days later, culture supernatants were collected to analyze the IgA, IgE, and IgG1 concentrations.

### B cell culture.

Mouse naive B cells were isolated from spleens with a Mouse B Cell Isolation Kit (Miltenyi Biotec). In all mouse B cell culture conditions, 500 ng/mL anti-mouse CD40 antibody was included. Mouse naive B cells were cultured in RPMI 1640 medium containing 10% FBS, 500 μg/mL penicillin, and 50 μM 2-mercaptoethanol with or without 0.8 nM bryostatin 1. For analysis of GLTα expression, mouse B cells were cultured with 5 ng/mL TGF-β for 48 hours. For analysis of PSTα and CTα expression, mouse B cells were cultured with 5 ng/mL TGF-β, 5 ng/mL IL-4, 5 ng/mL IL-5, 5 ng/mL BAFF, and 20 μg/mL LPS for 96 hours. For analysis of GLTε expression, mouse B cells were cultured with 5 ng/mL IL-4 for 48 hours. For PSTε and CTε expression analysis, mouse B cells were cultured with 5 ng/mL IL-4 for 96 hours. Human naive B cells were purchased from STEMCELL Technologies. In all human B cell culture conditions, 50 ng/mL MEGA CD40L was included. Human naive B cells were cultured in RPMI 1640 medium containing 10% FBS, 500 μg/mL penicillin, and 50 μM 2-mercaptoethanol with or without 800 nM bryostatin 1. To analyze GLTα1 and GLTα2 expression, human B cells were cultured with 5 ng/mL TGF-β for 48 hours. To analyze GLTε expression, human B cells were cultured with 5 ng/mL IL-4 for 48 hours. The inhibitors are listed in Table 2.

### Quantitative PCR.

RNA was isolated from cells with the Fast Gene RNA isolation kit (NIPPON Genetics), and single-stranded cDNA was synthesized by the GoScript Reverse Transcriptase (Promega) with the Random primer (Promega). Gene expression was analyzed by PCR using the KAPA SYBR Fast qPCR Kit (NIPPON Genetics). All PCR primer sequences are listed in Table 3.

### Western blot analysis.

Mouse naive B cells were isolated from the spleen with the Mouse B Cell Isolation Kit (Miltenyi Biotec) and cultured with the indicated stimulation for 1 hour. The cells were lysed by sonication in cell lysis buffer (1 M Tris-HCl pH 8.0, 1% NP-40 [Nacalai], 1:100 protease inhibitor cocktail [Nacalai], 1:100 phosphatase inhibitor cocktail [Nacalai]). Cell lysates were centrifuged for 15 minutes at 4°C. Supernatants were collected and boiled at 5 minutes at 95°C. These supernatants were separated by SDS-PAGE (Bio-Rad) and transferred to Amersham Protran Premium membrane (Cytiva). Blots were blocked with PhosphoBLOCKER (Cell Biolabs, Inc) for 1 hour at room temperature and reacted with the primary antibody diluted 1:1000 overnight at 4°C. Secondary antibodies were reacted 1 hour at room temperature. The signal was detected by Odyssey (LI-COR). The following antibodies were used: p-ERK (Cell Signaling Technology, 4370S, clone D13.14.4E), p-RUNX1(Affinity Biosciences, AF4316, polyclonal), ERK (Cell Signaling Technology, 4695S, clone 137F5), RUNX1 (Santa Cruz Biotechnology, sc-365644, polyclonal), PKC (Cell Signaling Technology, 2058S, polyclonal), ID2 (Origene, TA5001998, clone OTI9A8), α-tubulin (Medical & Biological Laboratories Co., PM054, polyclonal), goat anti-mouse IgG IRDye 800CW (LI-COR, 926-32210, polyclonal), and goat anti-rabbit IgG IRDye 800CW (LI-COR, 926-32211, polyclonal).

### RNA sequencing.

Mouse naive B cells were isolated from the spleen with the Mouse B Cell Isolation Kit (Miltenyi Biotec) and stimulated with or without bryostatin 1 in TGF-β– or IL-4–stimulated conditions. After 48 hours, B cells were harvested, and the total RNA was isolated with a Fast Gene RNA isolation kit (NIPPON Genetics). Libraries were prepared according to the manual using the MGIEasy RNA Directional Library Prep Set (MGI Tech). Circular DNA was generated by the MGIEasy Circularization Kit (MGI Tech). The DNA nanoball (DNB) was prepared by the DNBSEQ-G400RS High-throughput Sequencing Kit (MGI Tech). The generated DNB was sequenced using DNBSEQ G 400 under the condition of 2 × 100 bp. After removing the adaptor sequences in cutadapt (https://cutadapt.readthedocs.io/en/stable/), we used the sickle (https://sickle.readthedocs.io/en/latest/installation.html) to remove base pairs with a quality score of less than 20 and pair leads with a quality score of less than 40. Hisat2 (https://daehwankimlab.github.io/hisat2/) was used to map the sequenced read to the reference sequence and output the data in sam format. Samtools (https://anaconda.org/bioconda/samtools) was used to convert sam format to bam format, which was then sorted and indexed. featureCounts (https://subread.sourceforge.net/featureCounts.html) was used to count the reads that mapped to the genomic region of the reference sequence, followed by reads per kilobase million (RPKM) normalization and transcripts per million (TPM) to correct for the total number of reads and genomic length between samples. After normalization using the differential expression analysis from RNA-seq count data using robust normalization strategy (DEGES) normalization method of tag count comparison (TCC), edgeR (https://bioconductor.org/packages/release/bioc/html/edgeR.html) was used to identify differentially expressed genes.

### Statistics.

Samples were collected from 2–3 independent experiments (sample number is 3–5 in each experiment). Statistical analysis was performed using GraphPad Prism software. Data are plotted as mean ± SD. For comparison between 2 groups, an unpaired, 2-tailed Student’s *t* test was performed. For multiple comparisons, 1-way analysis of variance (ANOVA) was performed for groups with a normal distribution. *P* values are indicated in each figure.

### Study approval.

All experiments were performed following the guidelines of the Animal Care and Use Committee of Institute for Quantitative Biosciences, the University of Tokyo.

### Data availability.

All raw data from RNA-seq have been submitted to the NCBI Gene Expression Omnibus database repository (GEO GSE272975) and DDBJ Sequence Read Archive (accession number PRJDB37717 [PSUB043355]). The supplemental materials provide Supplemental Methods, Figures, Tables, and Supporting Data Values, including all quantitative data points used in graphs.

## Author contributions

NM and KY designed and performed the experiments. NM and RS planned and wrote the manuscript. NM, RT, PG, and RS discussed the data. TH, TI, and YY supported pathologic analysis. TN provided advice for animal experiments. TA, KIN, and MS provided the IgA-deficient, PKCδ-deficient, and ID2-deficient mice, respectively. MS and KIN provided experimental materials. HK provided a library of chemical compounds and advice for screening compounds. RS supervised this study.

## Funding support

- Platform Project for Supporting Drug Discovery and Life Science Research from the Japan Agency for Medical Research and Development (AMED) under grant number JP21am0101086 (support number 2628).
- AMED ACT-MS (JP16im0210608).
- Japan Society for the Promotion of Science Grant-in-Aid for Challenging Research (Pioneering/Exploratory).
- The Uehara Memorial Foundation.
- The Terumo Life Science Foundation.
- The Hoyu Science Foundation.
- UTOPIA.

## Supplementary Material

Supplemental data

Unedited blot and gel images

Supporting data values

## Figures and Tables

**Table 3:**
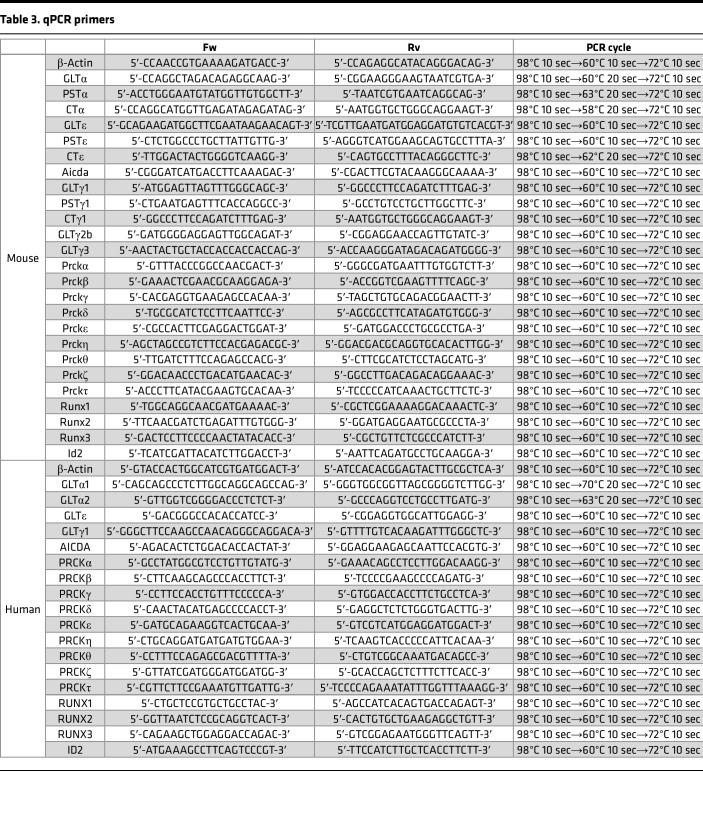
qPCR primers

**Table 2:**
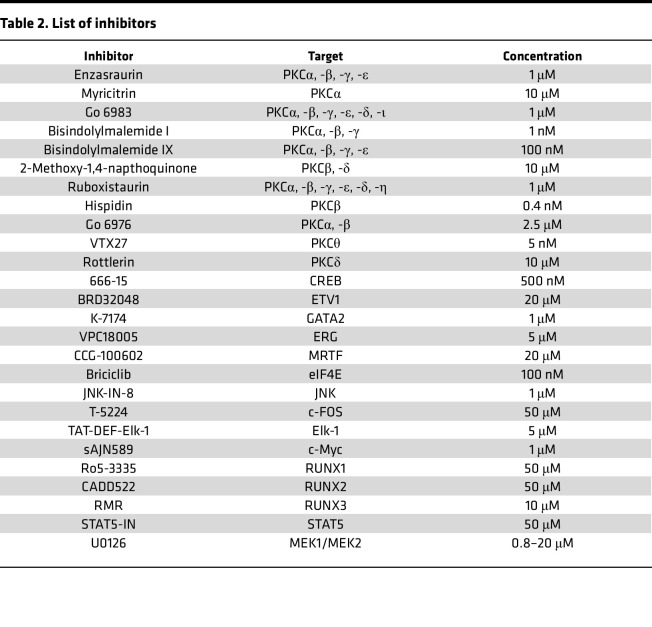
List of inhibitors

**Table 1:**
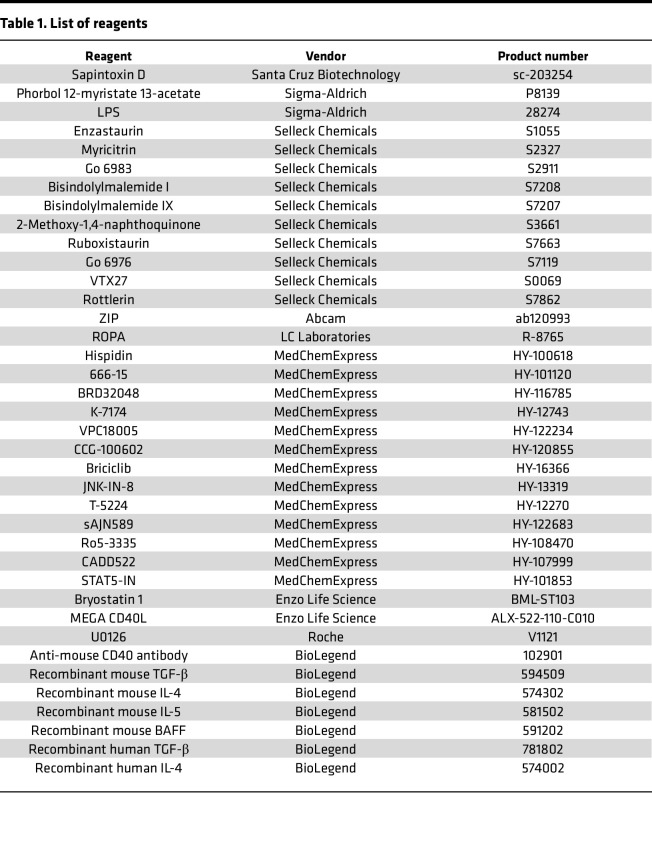
List of reagents

## Version 1 10/21/2025

In-Press Preview

## Version 2 12/08/2025

Electronic publication
