# Supplementary material for: Regulation of selective class switching provides long-term therapeutic benefits for hay fever
Source: JCI Insight. 2025 Oct 21;10(23):e190240. doi: 10.1172/jci.insight.190240 (PMC12890507; doi:10.1172/jci.insight.190240)
Supplement: Supplemental data [file jciinsight-10-190240-s012.pdf]

Supplemental Materials:

Regulation of selective class-switching provides long term therapeutic benefits for hay fever

Naoki Morita, Kohta Yamamoto, Ryutaro Tamano, Peng Gao, Takahiro Nagatake, Tianxiang Huang, Takenori Inomata, Yasuhiro Yamada, Takahiro Adachi, Manabu Sugai, Keiichi I. Nakayama, Hirotatsu Kojima, Reiko Shinkura

Corresponding author: rshinkura@iqb.u-tokyo.ac.jp

Supplemental Figures

Fig. S1

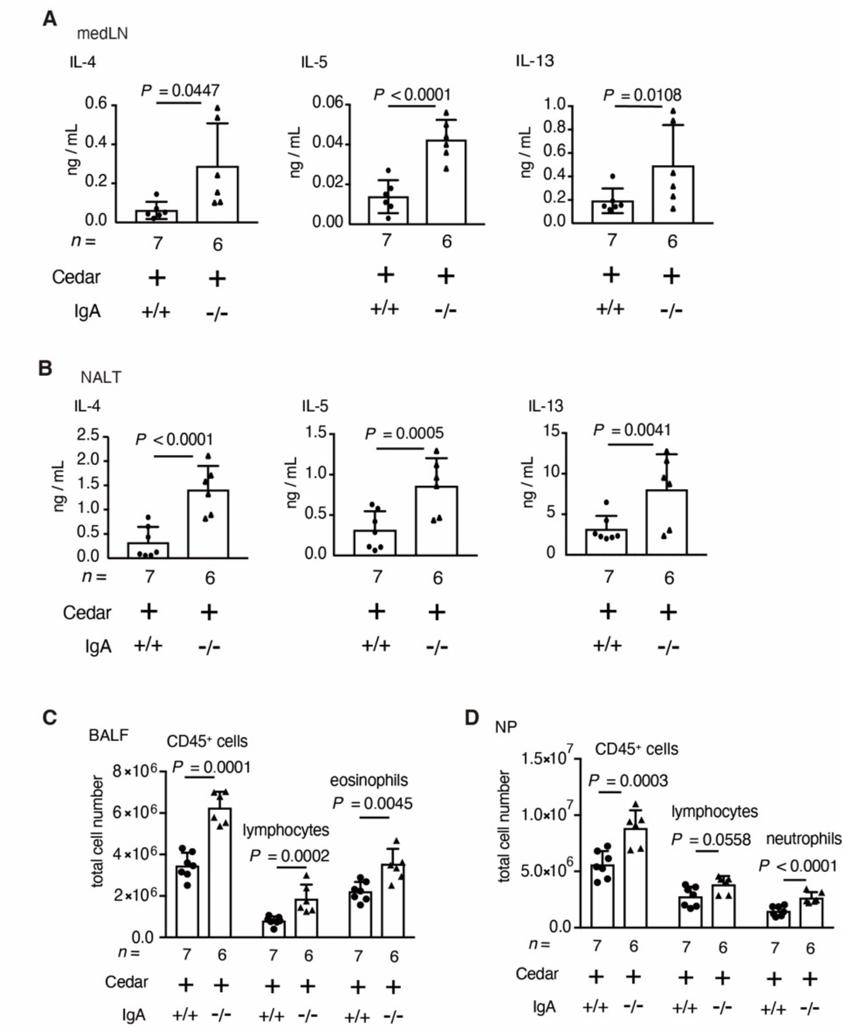

Supplemental Figure 1. Loss of IgA induces high susceptibility to cedar pollen-induced hay fever.

(A and B) Cytokine production from restimulated medLN cells and NALT cells with cedar pollen in an antigen-dependent manner (A: medLN, B: NALT) (n = 6-7). (C and D) The number of indicated immune cells in the BALF and nasal passage (NP) (C: BALF, D: NP) (n = 6-7). Statistical analysis was performed by unpaired Student's t test (A-D). Data are expressed as mean ± S.D. in (A-D).

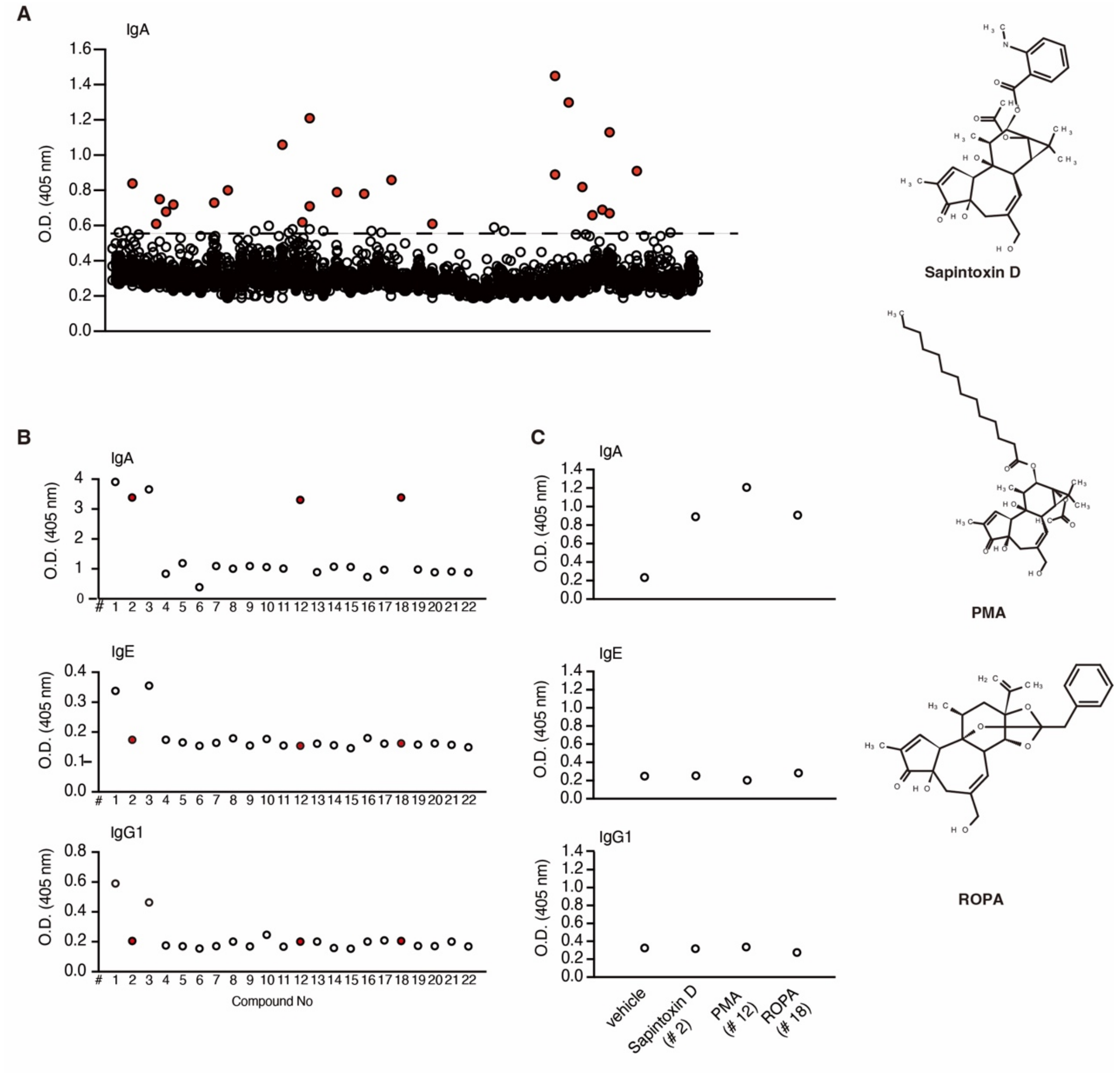

Supplemental Figure 2. Screening of IgA-inducible chemical compounds.

(A)1st screening of 3337 chemical compounds to identify IgA inducible molecule(s). (B)2nd screening of 22 chemical compounds to identify IgA, but not IgE and IgG1 inducible molecule(s). (C) 3rd screening of 3 chemical compounds to validate selective induction of IgA.

Fig. S3

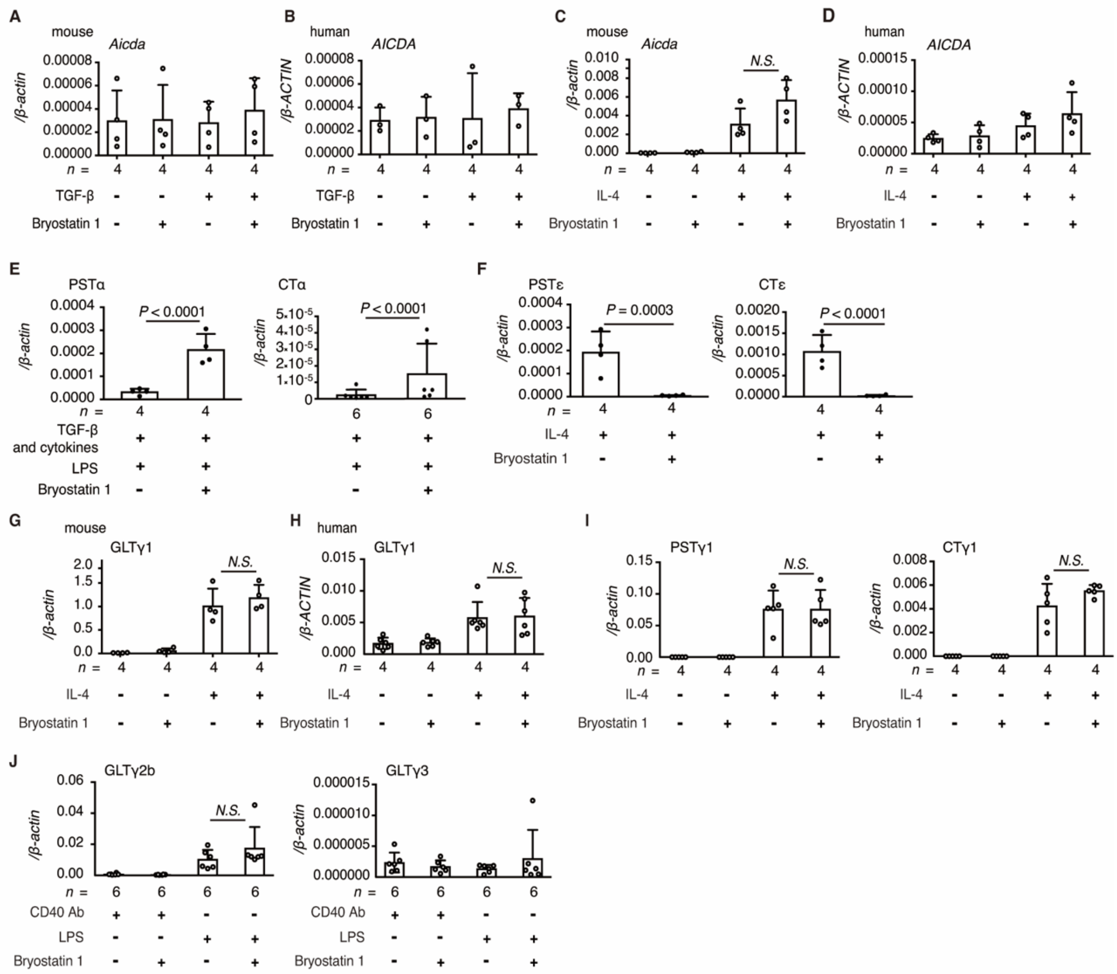

Supplemental Figure 3. Effect of Bryostatin 1 in AID expression and IgG class switching.

(A and C) Expression *Aicda* in cultured mouse B cells with indicated stimulation (*n* = 4). (B and D) Expression of *AICDA* in cultured human B cells with indicated stimulation (*n* = 4). (E) Expression of PSTα and CTα in cultured mouse B cells (n = 4-6). (F) Expression of PSTε and CTε in cultured mouse B cells (*n* = 4). (G-H) Expression of GLTγ1 in cultured (G) mouse and (H) human B cells with indicated stimulation (*n* = 4). (I) Expression of PSTγ1 and CTγ1 in cultured

mouse B cells with indicated stimulation (*n* = 4). (J) Expression of GLTγ2b and GLTγ3 in cultured mouse B cells with indicated stimulation (*n* = 6). Statistical analysis was performed by one-way ANOVA with Tukey's multiple comparisons test (A-D and G-J) and unpaired Student’s t test (E-F). Data are expressed as mean ± S.D. in (A-J).

Fig. S4

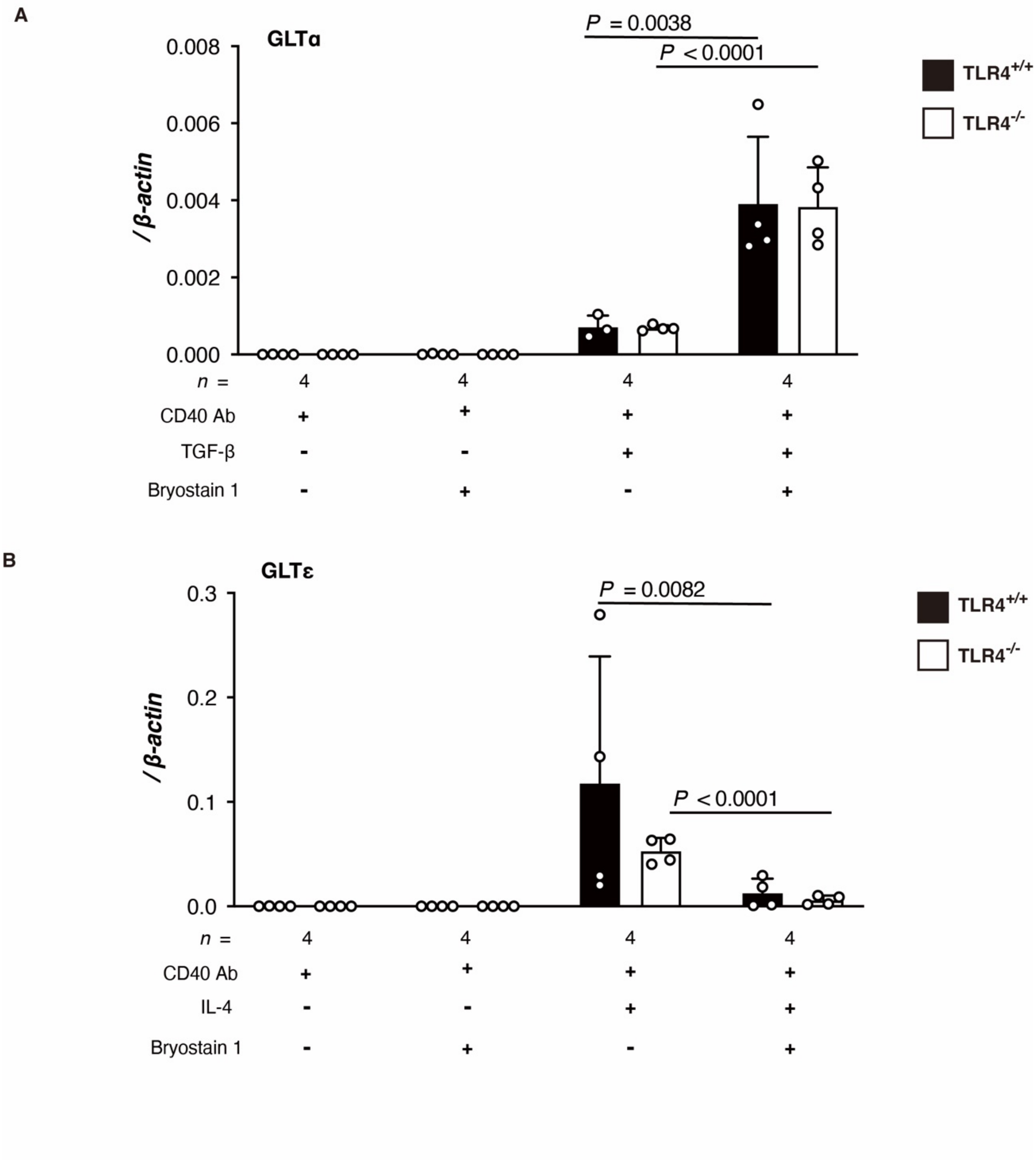

Supplemental Figure 4. TLR4 is not involved in Bryostatin 1-mediated regulation of GLT expression.

(A) Expression of GLTα in cultured mouse wild-type and *Tlr4* $^{-/-}$ B cells (n = 4).

(B) Expression of GLTε in cultured mouse wild-type and *Tlr4* $^{-/-}$ B cells (n = 4).

Statistical analysis was performed by one-way ANOVA with Tukey's multiple comparisons test (A-B). Data are expressed as mean ± s.d. in (A-B).

Fig. S5

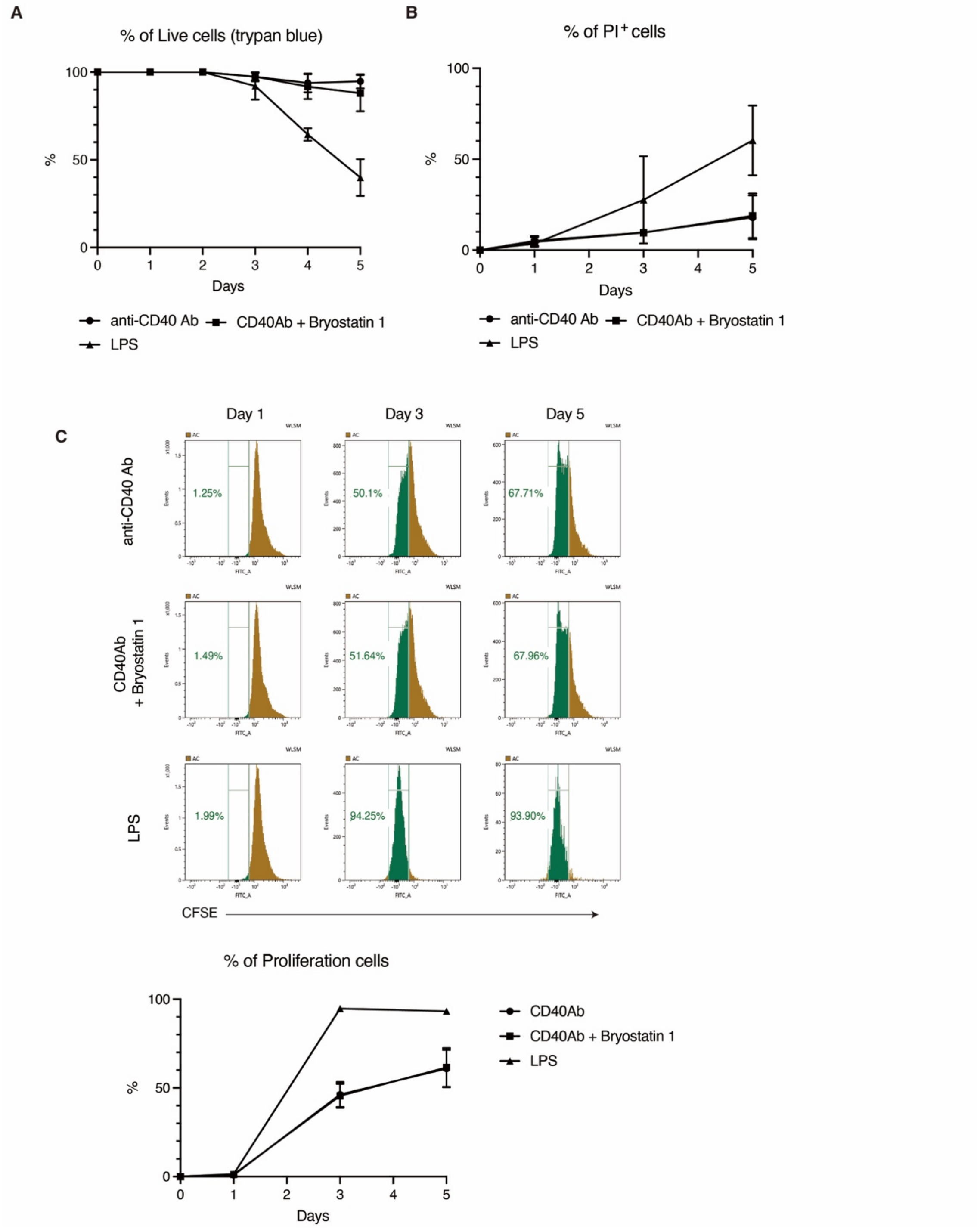

Supplemental Figure 5. Bryostatin 1 did not affect to the survival and proliferation of B cells.

(A)Ratio of living cells based on the trypan blue staining from day 0 to day 5 (n = 3). (B)Ratio of dying cells based on the PI staining from day 0 to day 5 (n = 3). (C) Representative FACS plot and ratio of proliferation cells based on the CFSE staining from day 0 to day 5 (n = 3).

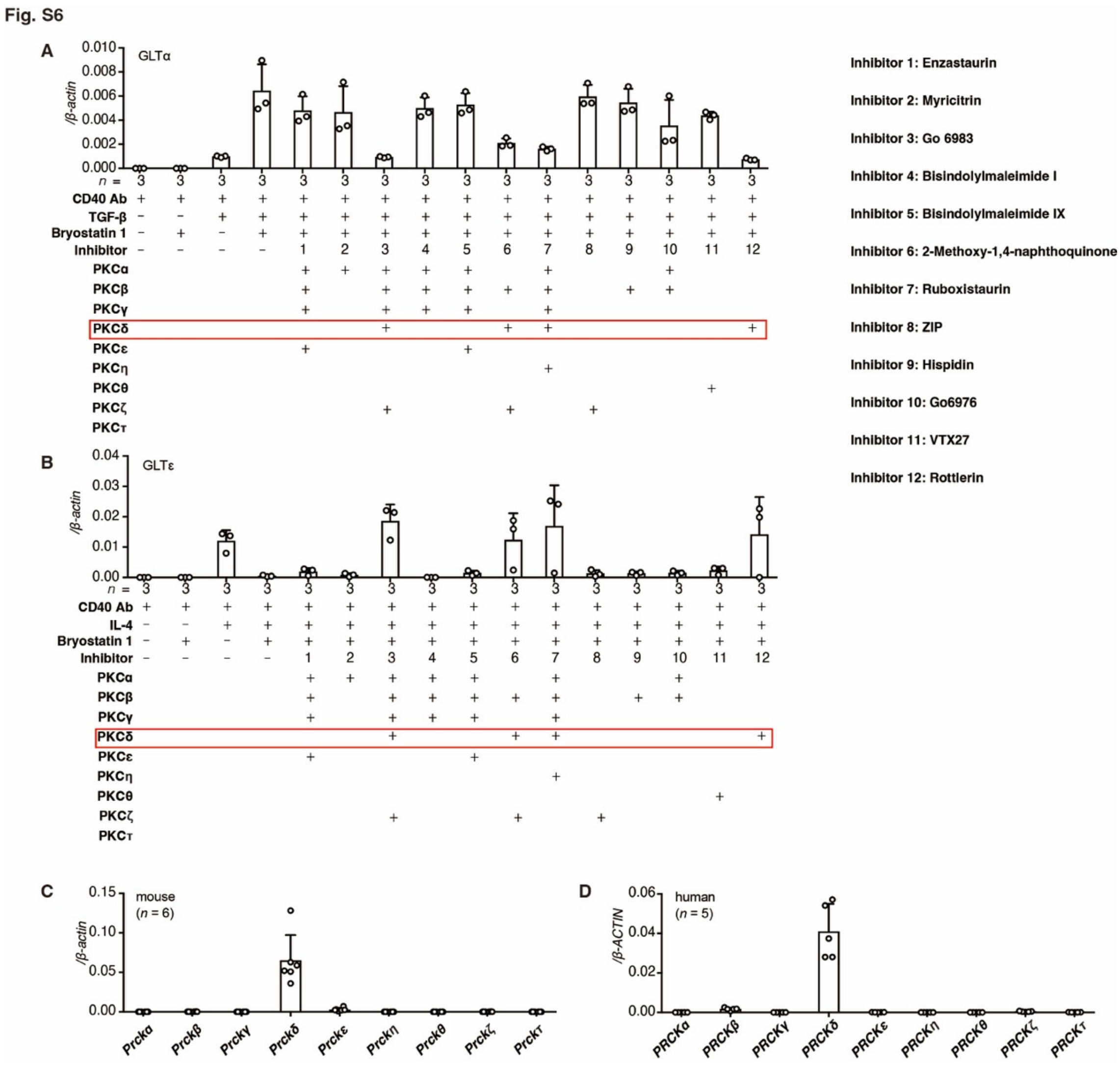

Supplemental Figure 6. Identification of Bryostatin 1-reacting PKC isozyme for regulation of class switching.

(A and B) Expression of GLTα and GLTε in cultured mouse B cells with indicated stimulation. Different spectra of PKC inhibitors were pretreated before stimulation of Bryostatin 1 (A: GLTα, B: GLTε) (n = 3). (C and D) Expression of PKC isozymes in isolated B cells (C: mouse n = 6, D: human n = 5). Data are expressed as mean ± S.D. in (A-D).

Fig. S7

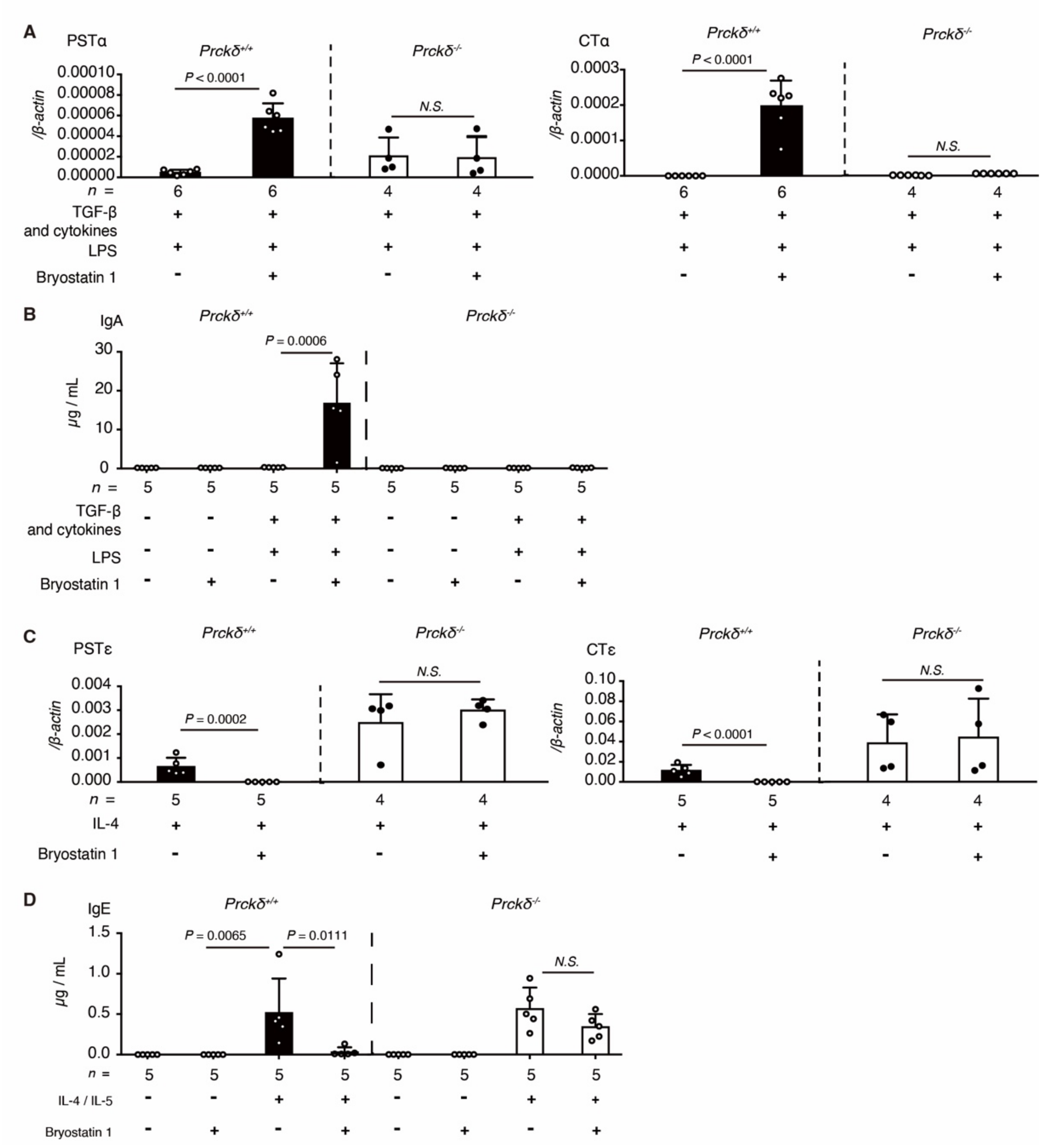

Supplementala Figure 7. Effect of Bryostatin 1 in expression of class switching-related transcripts and production of antibody

(A) Expression of PSTα and CTα in cultured mouse wild-type (n = 6) and *Prckd* $^{-/-}$ (n = 4) B cells. (B) Production of IgA in the supernatant of cultured wild-type and *Prckd* $^{-/-}$ B cells (n = 5). (C) Expression of PSTε and CTε in cultured

mouse wild-type (n = 5) and *Prckd* $^{-/-}$ (n = 4) B cells. (D) Production of IgE in the supernatant of cultured wild-type and *Prckd* $^{-/-}$ B cells (n = 5). Statistical analysis was performed by unpaired Student’s t test (A and C) and one-way ANOVA with Tukey's multiple comparisons test (B and D). Data are expressed as mean ± S.D. in (A-D).

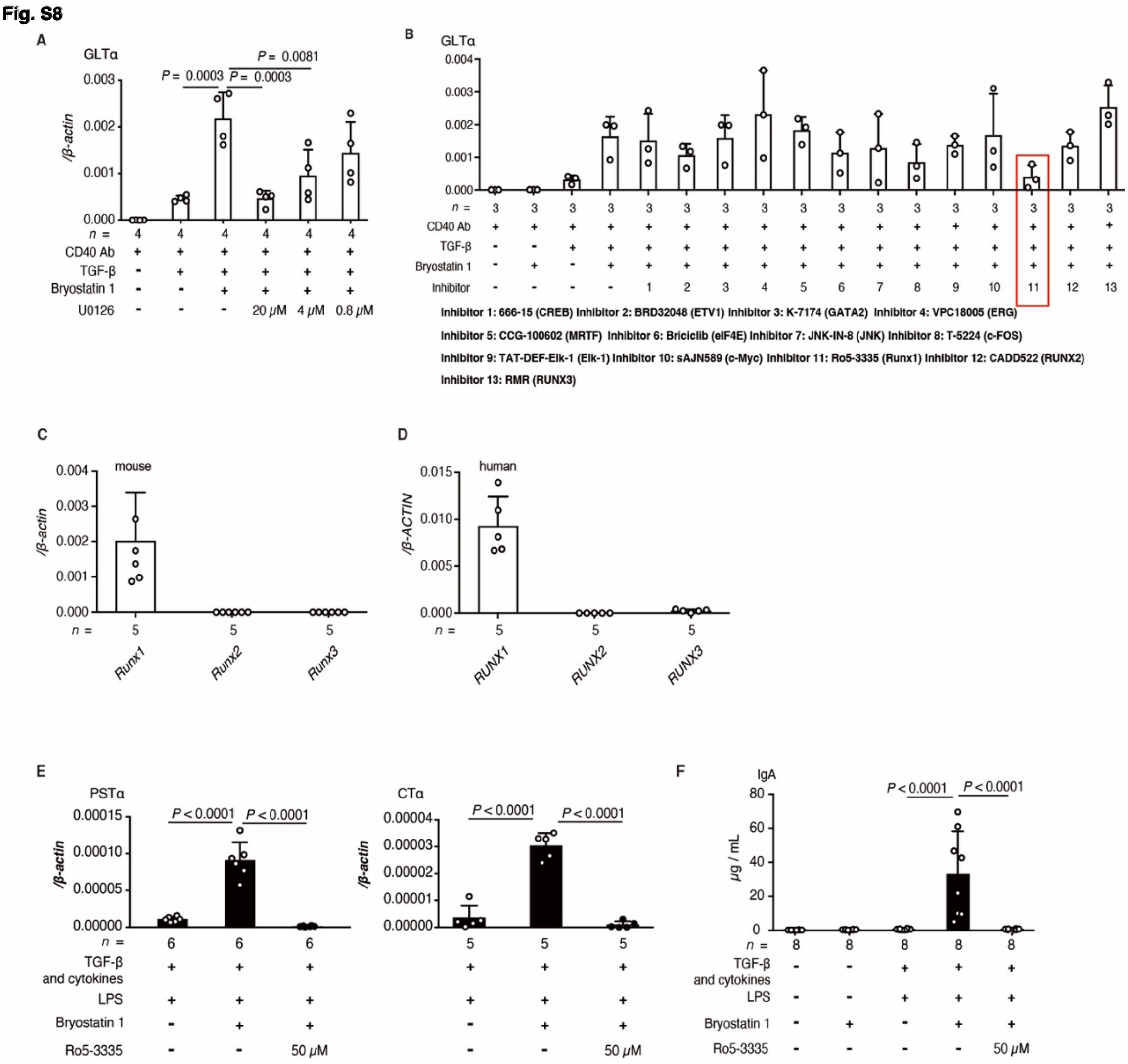

Supplemental Figure 8. Identification of Bryostatin 1-reacting transcription factor for expression of GLTα.

(A) Expression of GLTα in cultured mouse B cells with indicated stimulation. MEK1/2 inhibitor (U0126) was pretreated with indicated concentrations before stimulation of Bryostatin 1 ($n = 4$). (B) Expression of GLTα in cultured mouse B cells with indicated stimulation ($n = 3$). Inhibitors of transcription factor in downstream signaling of ERK1/2 were pretreated before stimulation of

Bryostatin 1. (C and D) Expression of RUNX family in isolated B cells (C: mouse $n = 5$, D: human $n = 5$). (E) Expression of PSTα ($n = 6$) and CTα ($n = 5$) in cultured mouse B cells with or without Ro5-3335. (F) Production of IgA in the supernatant of cultured B cells ($n = 8$). RUNX1 inhibitor was pretreated before stimulation of Bryostatin 1. Statistical analysis was performed by one-way ANOVA with Tukey's multiple comparisons test (A and E-F). Data are expressed as mean ± S.D. in (A-F).

Fig. S9

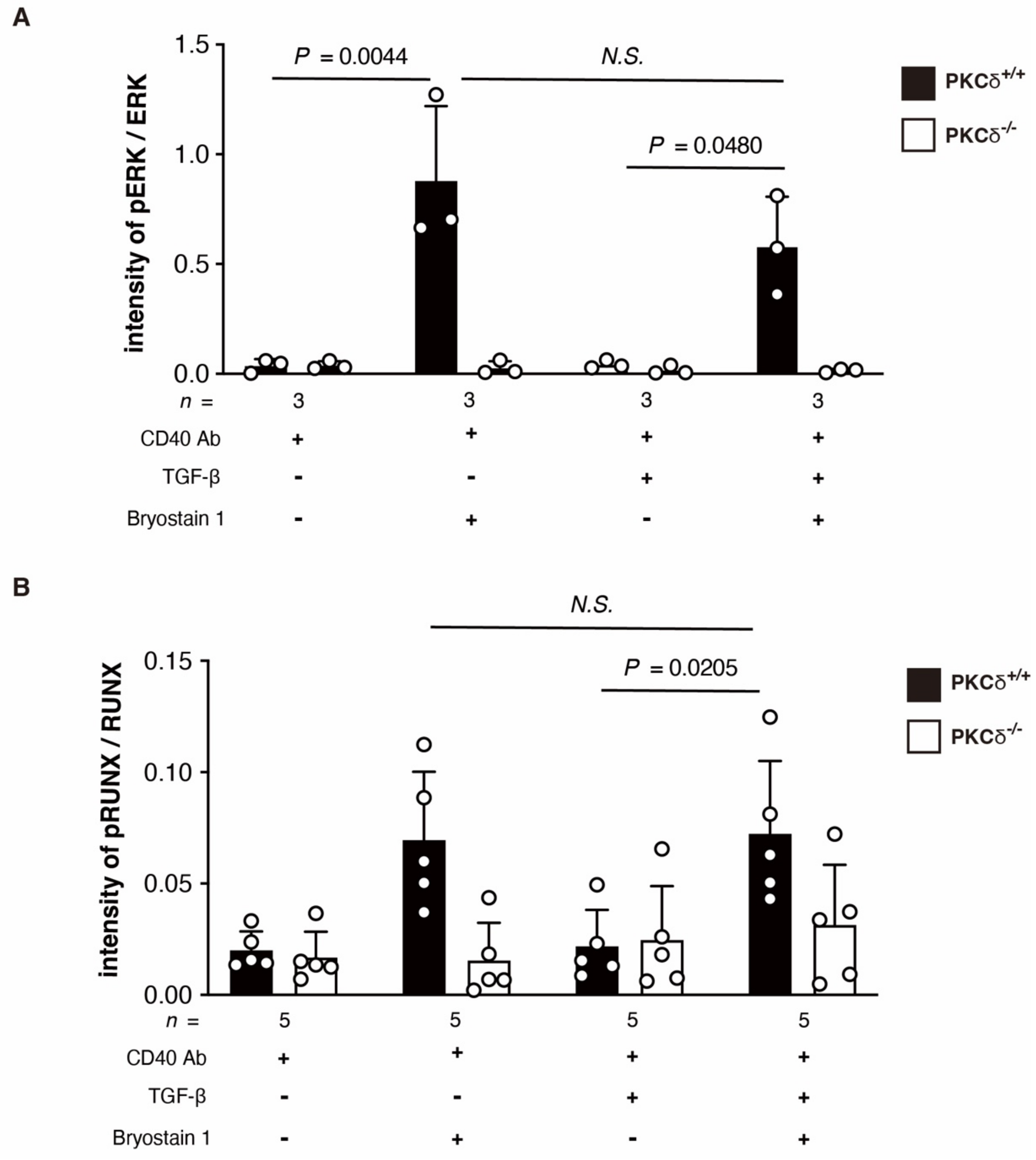

Supplemental Figure 9. Bryostatin 1 induces IgA class-switching through ERK and RUNX1 phosphorylation.

(A) Band intensity was determined by Odyssey imaging system. Ratio of phosphorylated ERK and ERK intensity (n = 3). (B) Ratio of phosphorylated RUNX1 and RUNX1 intensity (n = 5). Statistical analysis was performed by one-

way ANOVA with Tukey's multiple comparisons test (A-B). Data are expressed as mean ± s.d. in (A-B).

Fig. S10

A

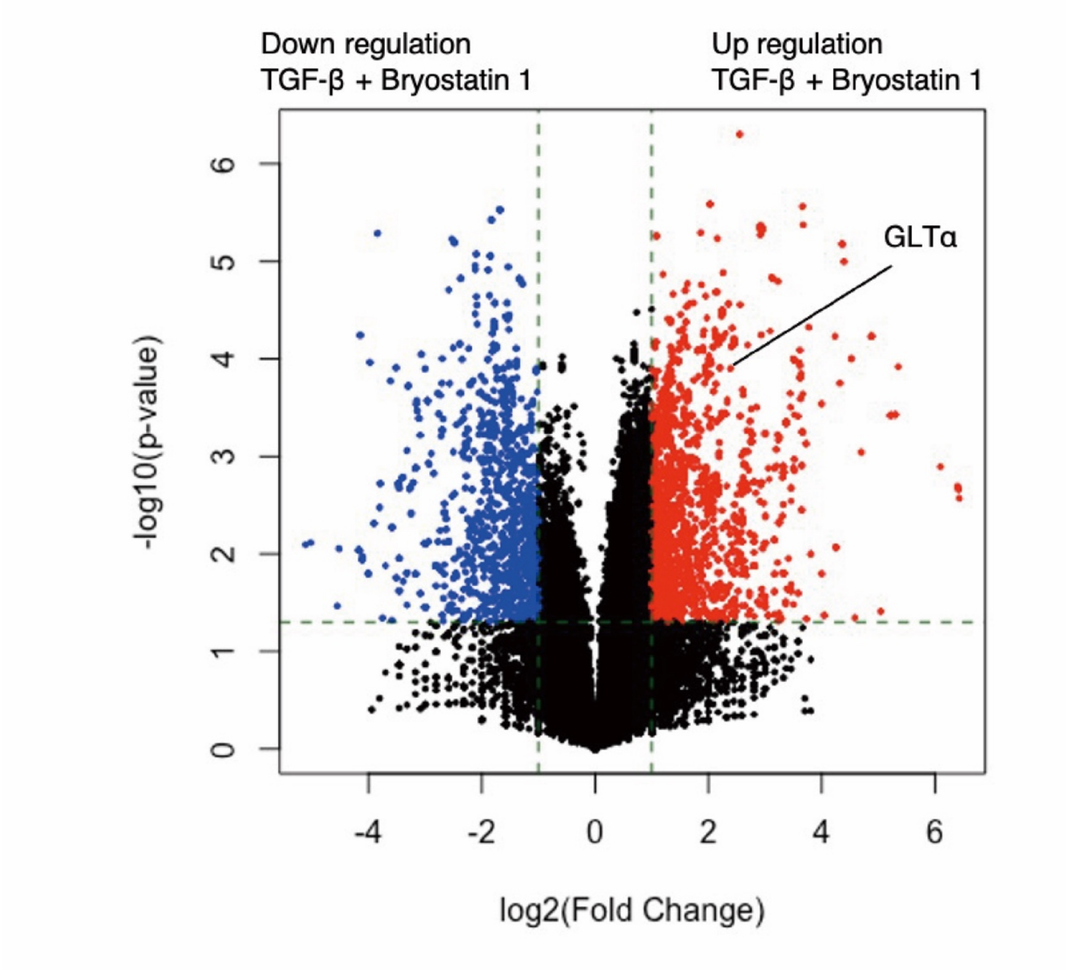

Supplemental Figure 10. Treatment of Bryostatin 1 show different type gene expression pattern compared with TGF-*β* alone.

(A)Volcano plot of differentially expressed genes in bulk RNA sequencing of TGF-β stimulated B cells cultured with or without Bryostatin 1.

Fig. S11

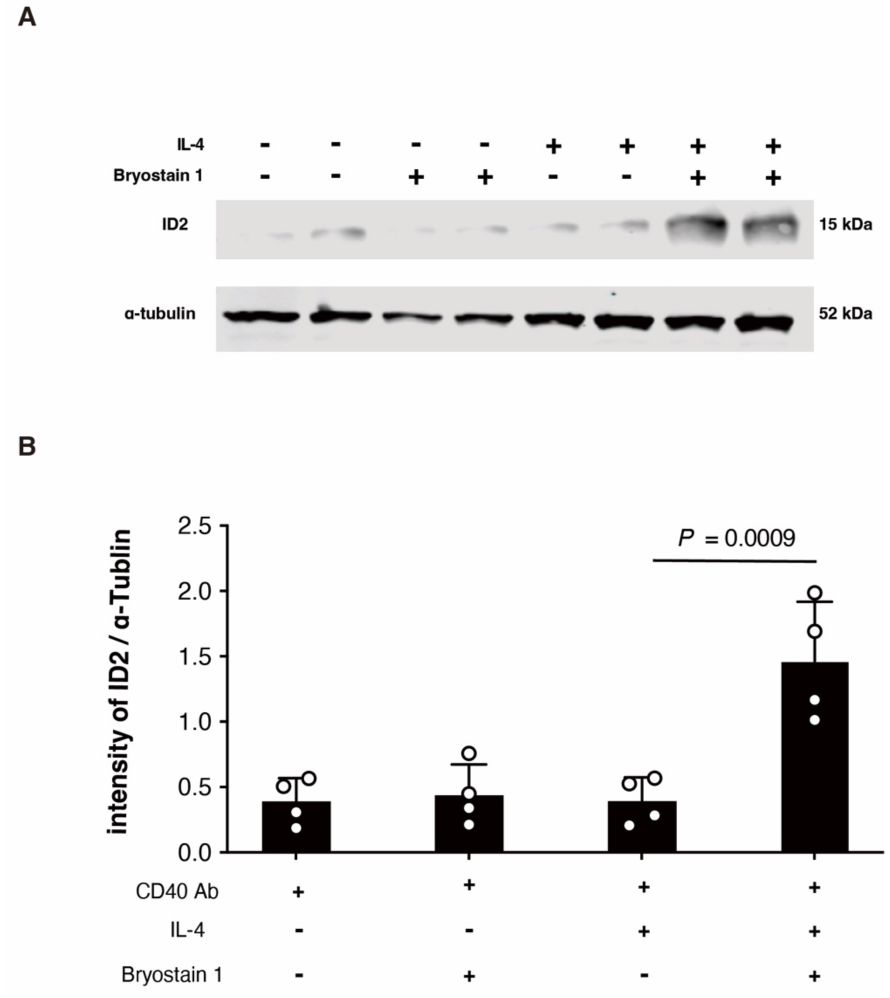

Supplemental Figure 11. Bryostatin 1 induces ID2 expression at protein level.

(A)Representative image of Immunoblot analysis of ID2 and α-tubulin in B cells. (B) Band intensity was determined by Odyssey imaging system. Ratio of ID2 and α-tubulin intensity. Statistical analysis was performed by one-way ANOVA with Tukey's multiple comparisons test (n = 4). Data are expressed as mean ± s.d. in (B).

129

Fig. S12

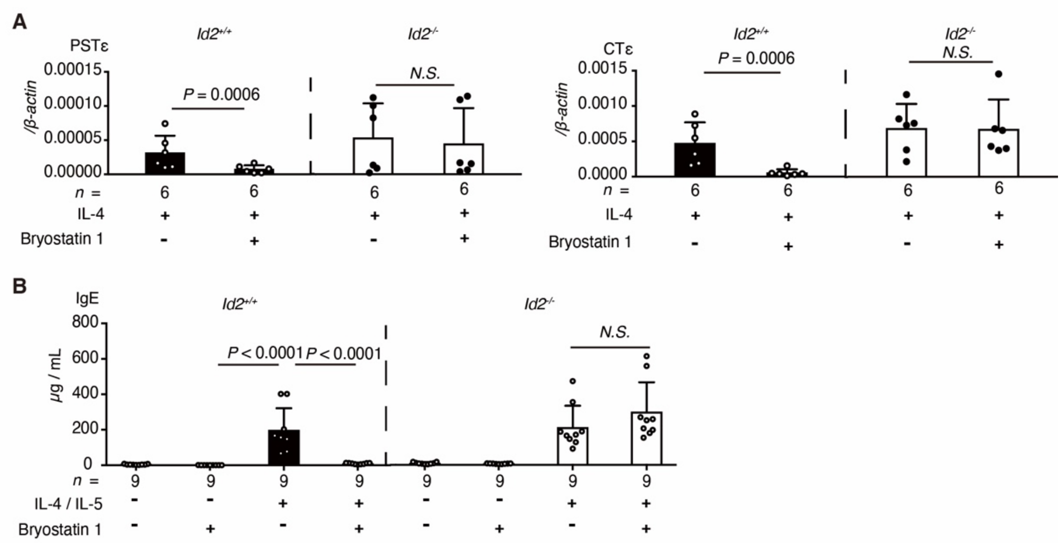

Supplemental Figure 12. Bryostatin 1 suppresses IgE class-switching through ID2 expression.

(A) Expression of PSTε and CTε in cultured wild-type and *Id2* $^{-/-}$ (*n* = 6) B cells. (B) Production of IgE in the supernatant of cultured wild-type and *Id2* $^{-/-}$ B cells (*n* = 9). Statistical analysis was performed by unpaired Student's t test (A) and one-way ANOVA with Tukey's multiple comparisons test (B). Data are expressed as mean ± S.D. in (A-B).

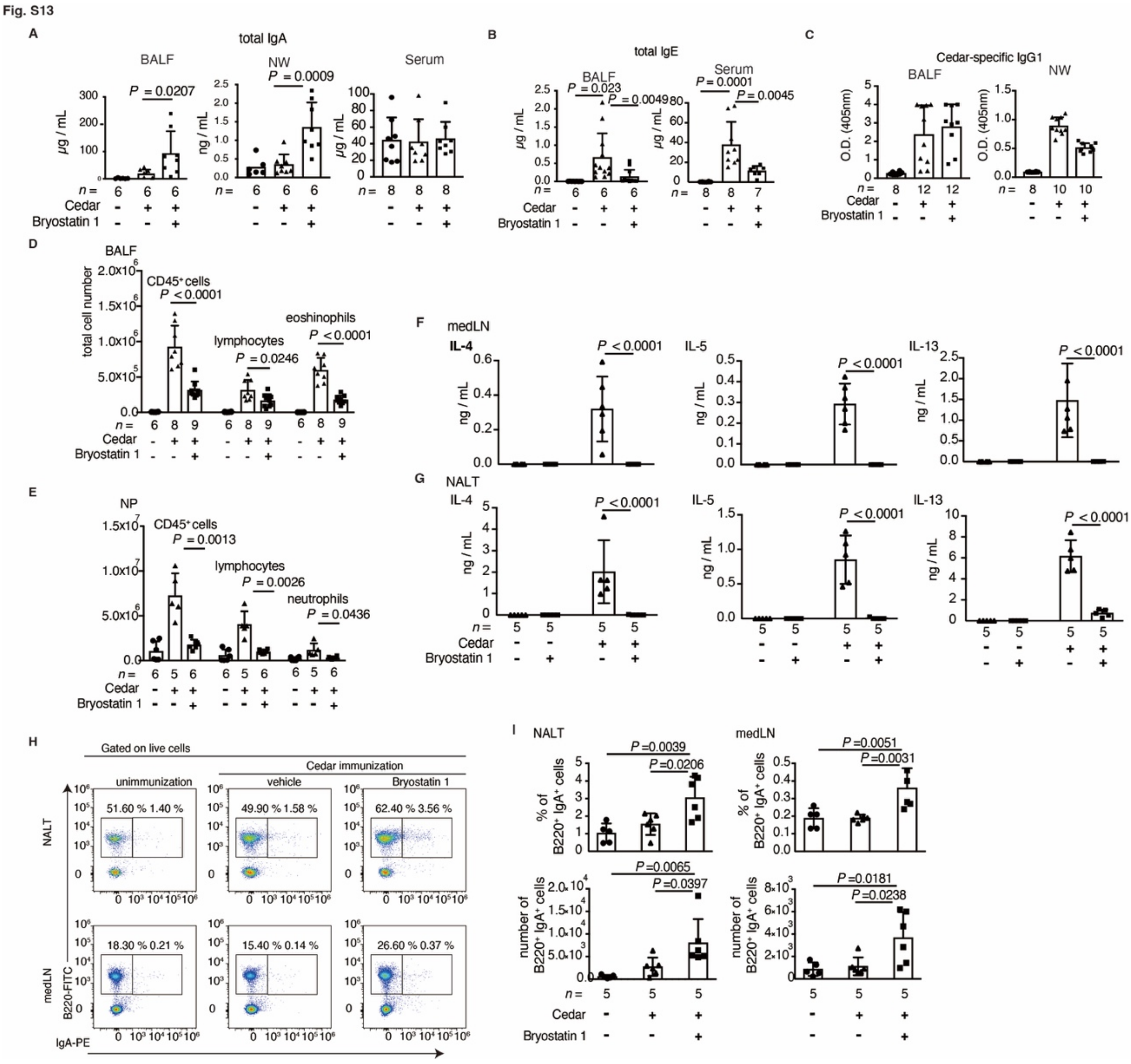

Supplemental Figure 13. Bryostatin 1 ameliorates cedar pollen-induced hay fever.

(A) Total IgA in the BALF, NW and serum (*n* = 7-10). (B) Total IgE in the BALF and serum (*n* = 7-9). (C) Cedar pollen-specific IgG1 in the BALF and NW (*n* = 8-12). (D and E) The number of indicated immune cells in the BALF and NP (D: BALF, E: NP) (*n* = 5-9). (F and G) Cytokines production from restimulated medLN cells and NALT cells with cedar pollen (F: medLN, G: NALT) (*n* = 5). (H)

Representative plots of IgA^+ $B220^+$ cells from NALT and medLN in indicated *in vivo* condition. (I) Frequency and absolute number of IgA^+ $B220^+$ cells in the NALT and medLN (n = 5). Statistical analysis was performed by one-way ANOVA with Tukey's multiple comparisons test (A-E and I) or unpaired Student’s t test (F-G). Data are expressed as mean ± S.D. in (A-G and I).

Fig. S14

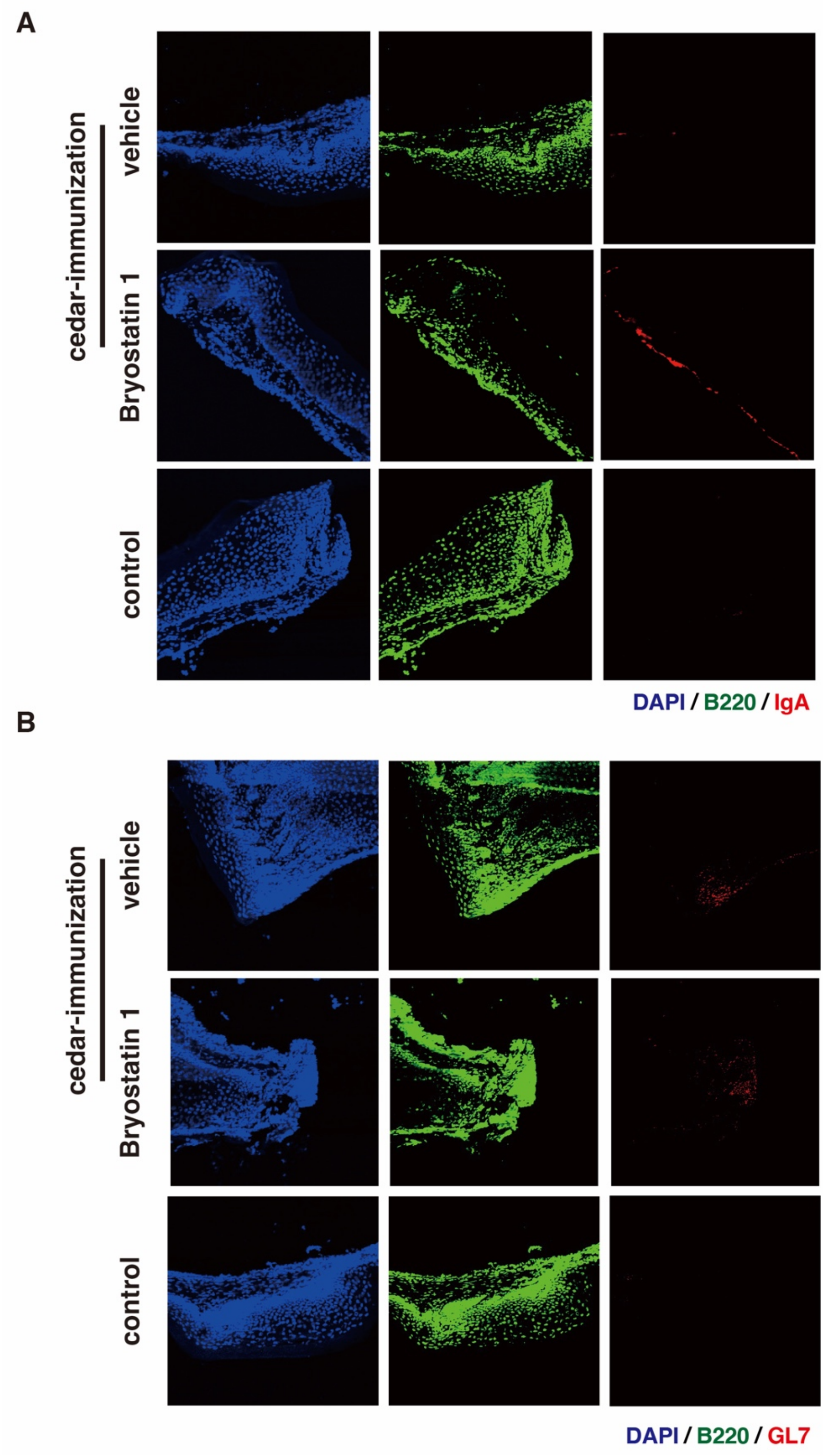

Supplemental Figure 14 Bryostatin 1 induce IgA^{+} B cells, but does not increased germinal center (GC) B cells in the NALT.

(A) Representative images show the IgA$^+$ B cells in the NALT stained with IgA (red), B220 (green) and DAPI (blue). (B) Representative images show the GC areas in the NALT stained with GL7 (red), B220 (green) and DAPI (blue).

Fig. S15

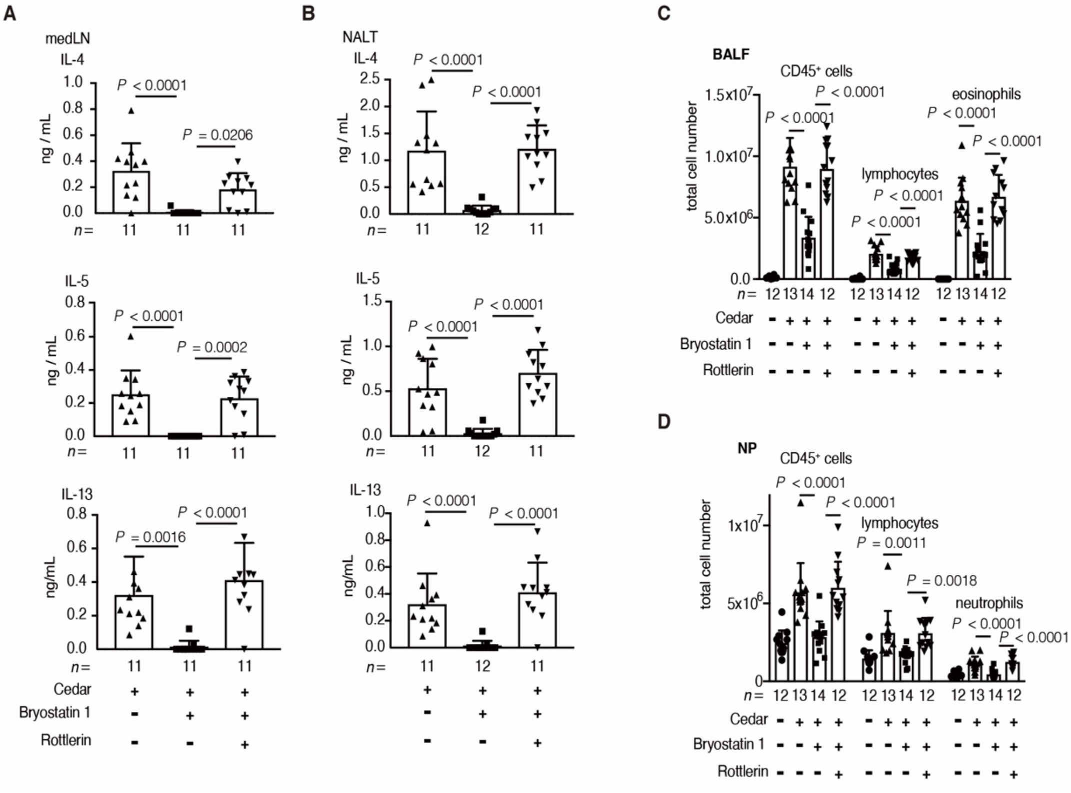

Supplemental Figure 15. Loss PKCδ signaling induces high susceptibility to cedar pollen-induced hay fever.

(A and B) Cytokine production from restimulated medLN cells and NALT cells with cedar pollen (A: medLN, B: NALT) (*n* = 11-12). (C and D) The number of indicated immune cells in the BALF and NP (C: BALF, D: NP) (*n* = 12-14). Statistical analysis was performed by one-way ANOVA with Tukey's multiple comparisons test (A-D). Data are expressed as mean ± S.D. in (A-D).

Fig. S16

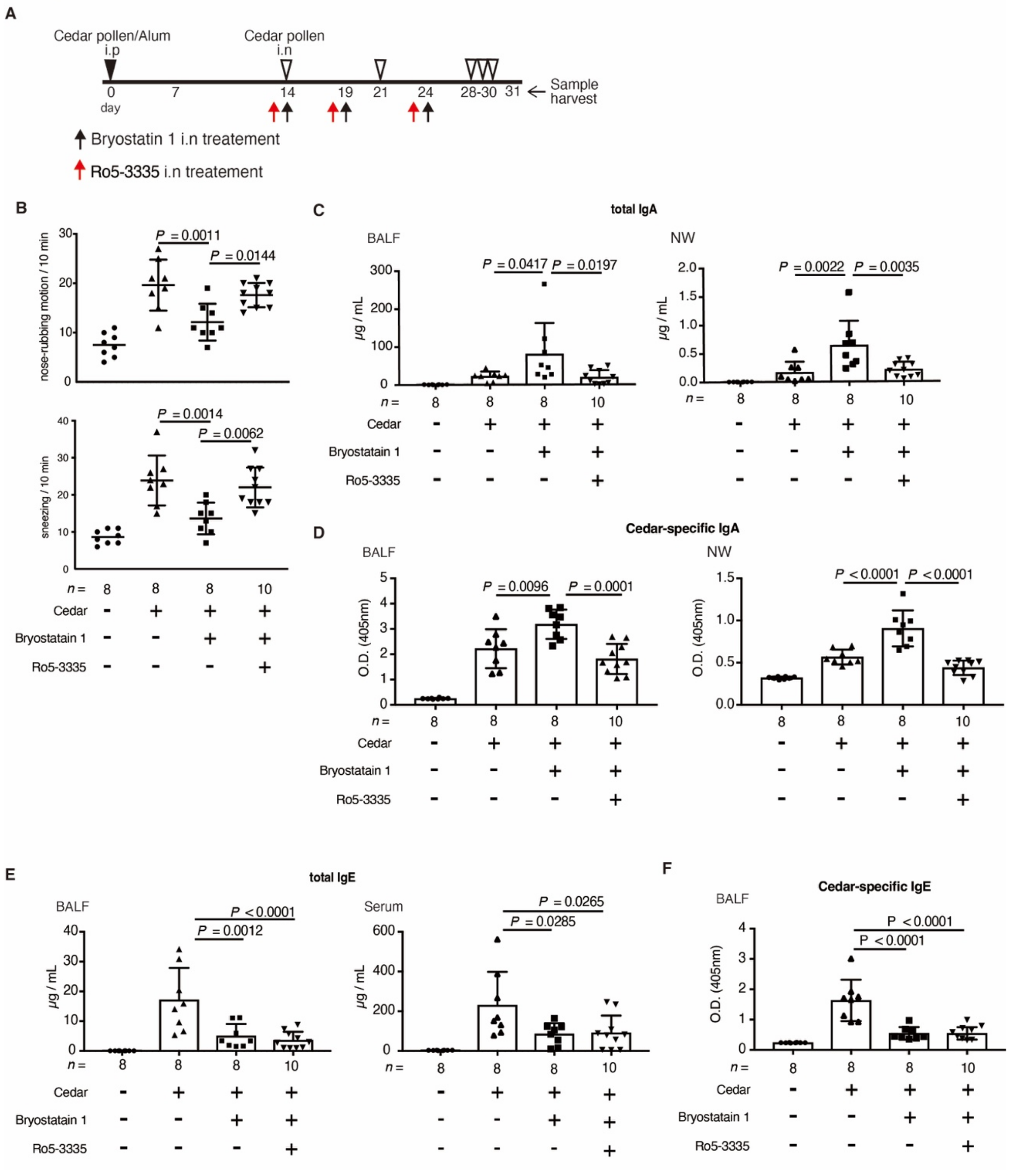

Supplemental Figure 16 Enhancement of IgA production induced by Bryostatin 1 in a RUNX1-dependent manner.

(A) A scheme of cedar pollen-induced hay fever model mice with or without treatment with RUNX1 inhibitor (Ro5-3335). (B) The rate of nose-rubbing

motion and sneezing mediated by the administration of cedar pollen via the intranasal route (n = 8-10). (C-D) Total and cedar pollen-specific IgA (C: total, D: cedar pollen-specific) (n = 10). (E-F) Total and cedar pollen-specific IgE (D: total, E: cedar pollen-specific) (n = 10). Statistical analysis was performed by one-way ANOVA with Tukey's multiple comparisons test (B-F). Data are expressed as mean ± s.d. in (B-E).

Fig. S17

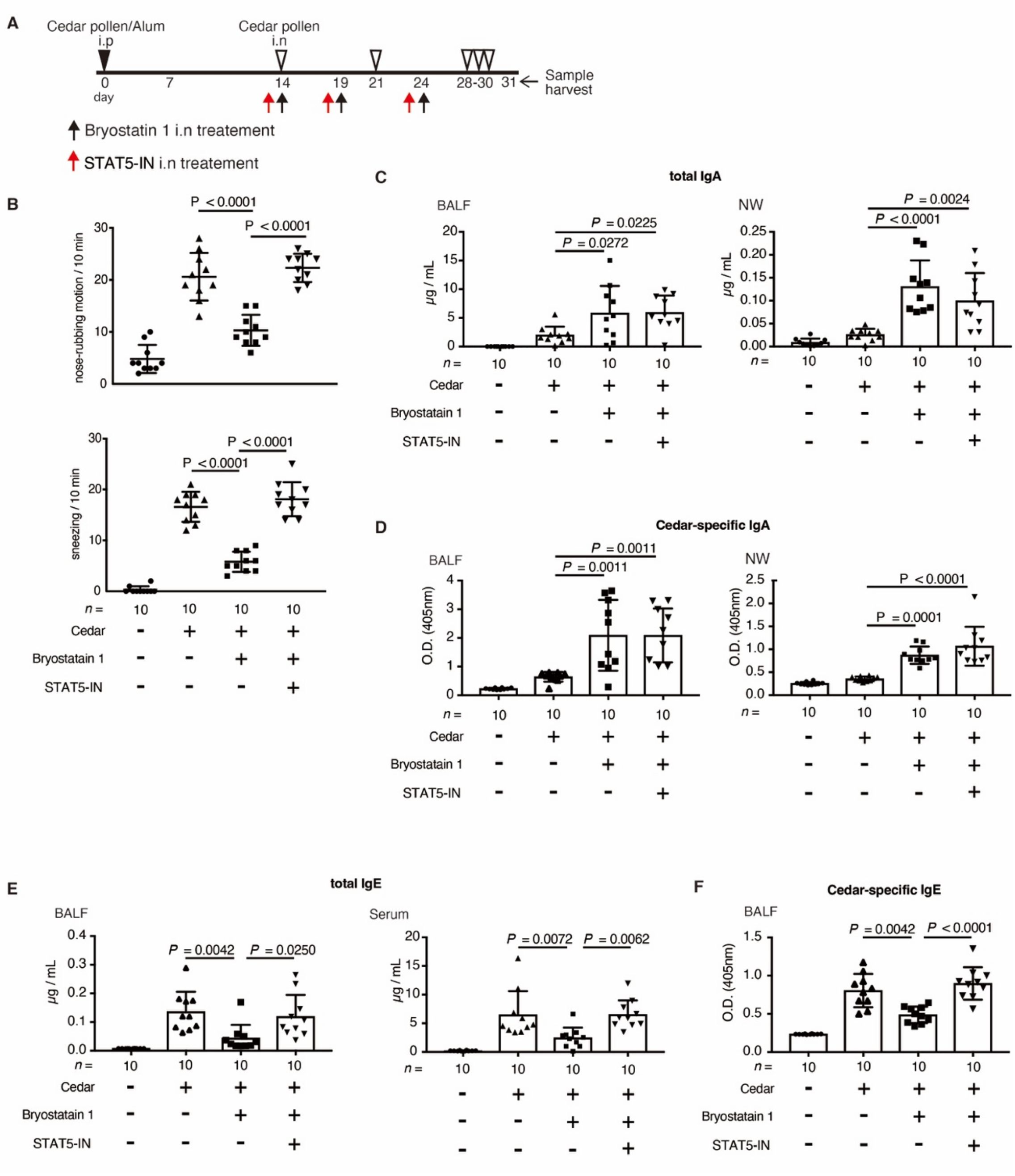

Supplemental Figure 17 Suppression of IgE production induced by Bryostatin 1 in a STAT5-dependent manner.

(A) A scheme of the study in cedar pollen-induced hay fever model mice with or without treatment with STAT5 inhibitor (STAT5-IN). (B) The rate of nose-rubbing

motion and sneezing mediated by the administration of cedar pollen via the intranasal route (n = 12). (C and D) Total and cedar pollen-specific IgA (C: total, D: cedar pollen-specific) (n = 12-13). (E-F) Total and cedar pollen-specific IgE (E: total, F: cedar pollen-specific) (n = 12-13). Statistical analysis was performed by one-way ANOVA with Tukey's multiple comparisons test (B-F). Data are expressed as mean ± S.D. in (B-F).

Fig. S18

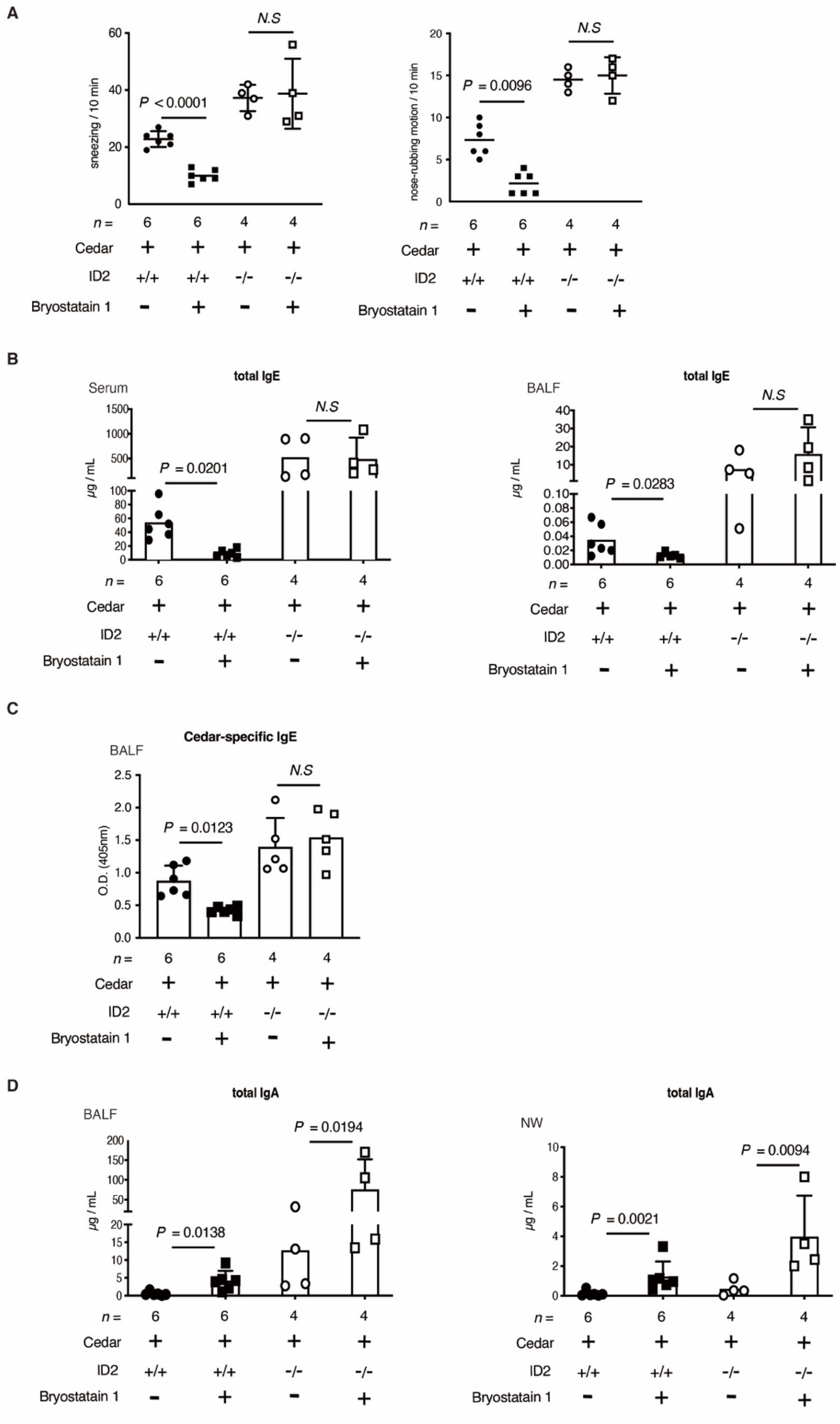
A
sneezing / 10 min
P < 0.0001
N.S
nose-rubbing motion / 10 min
P = 0.0096
n = 6 6 4 4
Cedar + + + +
ID2 +/+ +/+ -/- -/-
Bryostatain 1 - + - +
B
total IgE
Serum
BALF
μg / mL
P = 0.0201
P = 0.0283
C
Cedar-specific IgE
BALF
O.D. (405nm)
P = 0.0123
D
total IgA
BALF
NW
P = 0.0138
P = 0.0194
P = 0.0021
P = 0.0094

Supplemental Figure 18 Suppression of IgE production induced by Bryostatin 1 in an ID2-dependent manner.

(A) The rate of nose-rubbing motion and sneezing mediated by the administration of cedar pollen via the intranasal route in wild-type and Id2-deficient mice (*n* = 4-6). (B-C) Total and cedar pollen-specific IgE (B: total, C: cedar pollen-specific) (*n* = C). (D) Total IgA (D: total, E: cedar pollen-specific) (*n* = 4-6). Statistical analysis was performed by one-way ANOVA with Tukey's multiple comparisons test (B-D). Data are expressed as mean ± s.d. in (B-D).

Fig S19

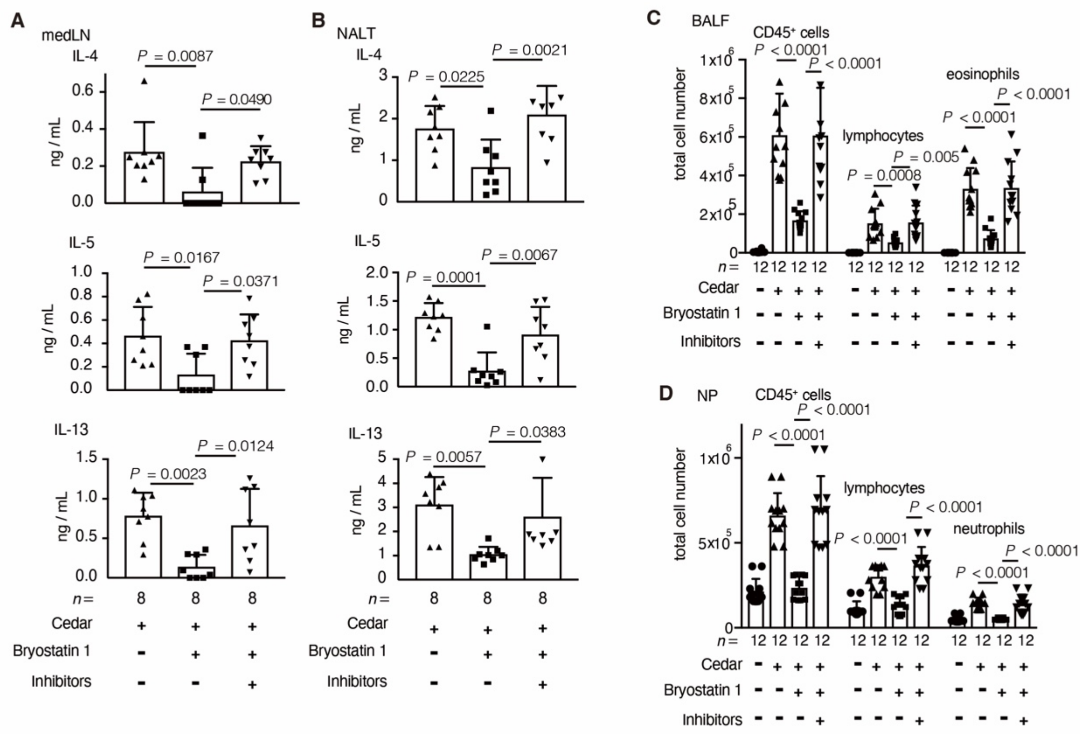

Supplemental Figure 19. Loss RUNX1 and STAT5 signaling induces high susceptibility to cedar pollen-induced hay fever.

(A and B) Cytokine production from restimulated medLN cells and NALT cells with cedar pollen (A: medLN, B: NALT) (n = 8). (C and D) The number of indicated immune cells in the BALF and NP (C: BALF, D: NP) (n = 12). Statistical analysis was performed by one-way ANOVA with Tukey's multiple comparisons test (A-D). Data are expressed as mean ± S.D. in (A-D).

Fig S20

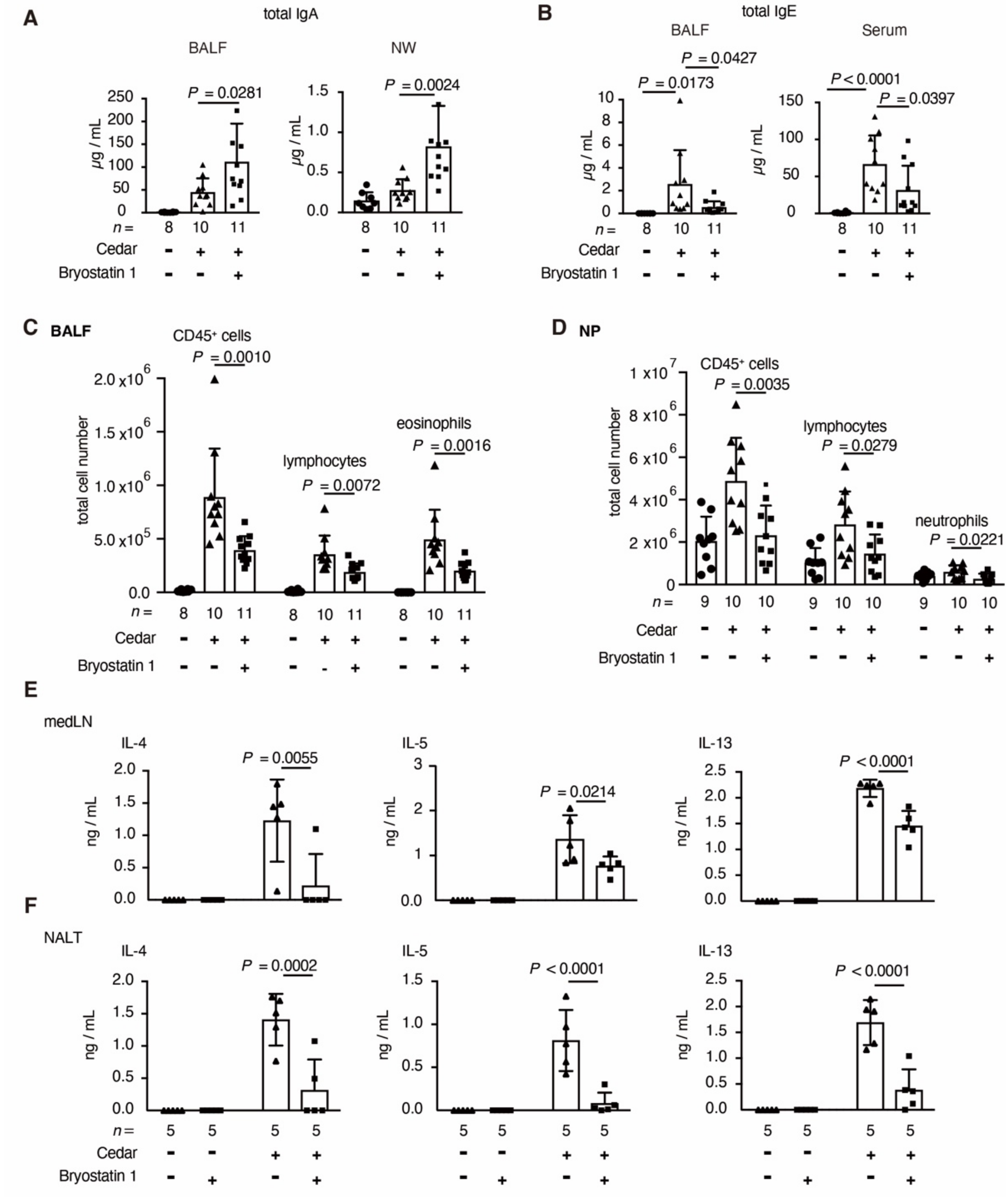

Supplemental Figure 20. Bryostatin 1 attenuated Th2 responses in cedar pollen-induced seasonal exposure hay fever model.

(A) Total IgA in the BALF and NW (*n* = 8-11). (B) Total IgE in the BALF and serum (*n* = 8-11). (C and D) The number of indicated immune cells in the BALF and NP (C: BALF, D: NP) (*n* = 8-11) (E and F) Cytokines production from

restimulated medLN cells and NALT cells with cedar pollen (E: medLN, F: NALT) (*n* = 5). Statistical analysis was performed by one-way ANOVA with Tukey's multiple comparisons test (A-D) or unpaired Student’s t test (E-F). Data are expressed as mean ± S.D. in (A-F).
