# Supplementary material for: Regulation of selective class switching provides long-term therapeutic benefits for hay fever
Source: JCI Insight. 2025 Oct 21;10(23):e190240. doi: 10.1172/jci.insight.190240 (PMC12890507; doi:10.1172/jci.insight.190240)

Full unedited gel for Figure 3

α-tubulin
52 kDa

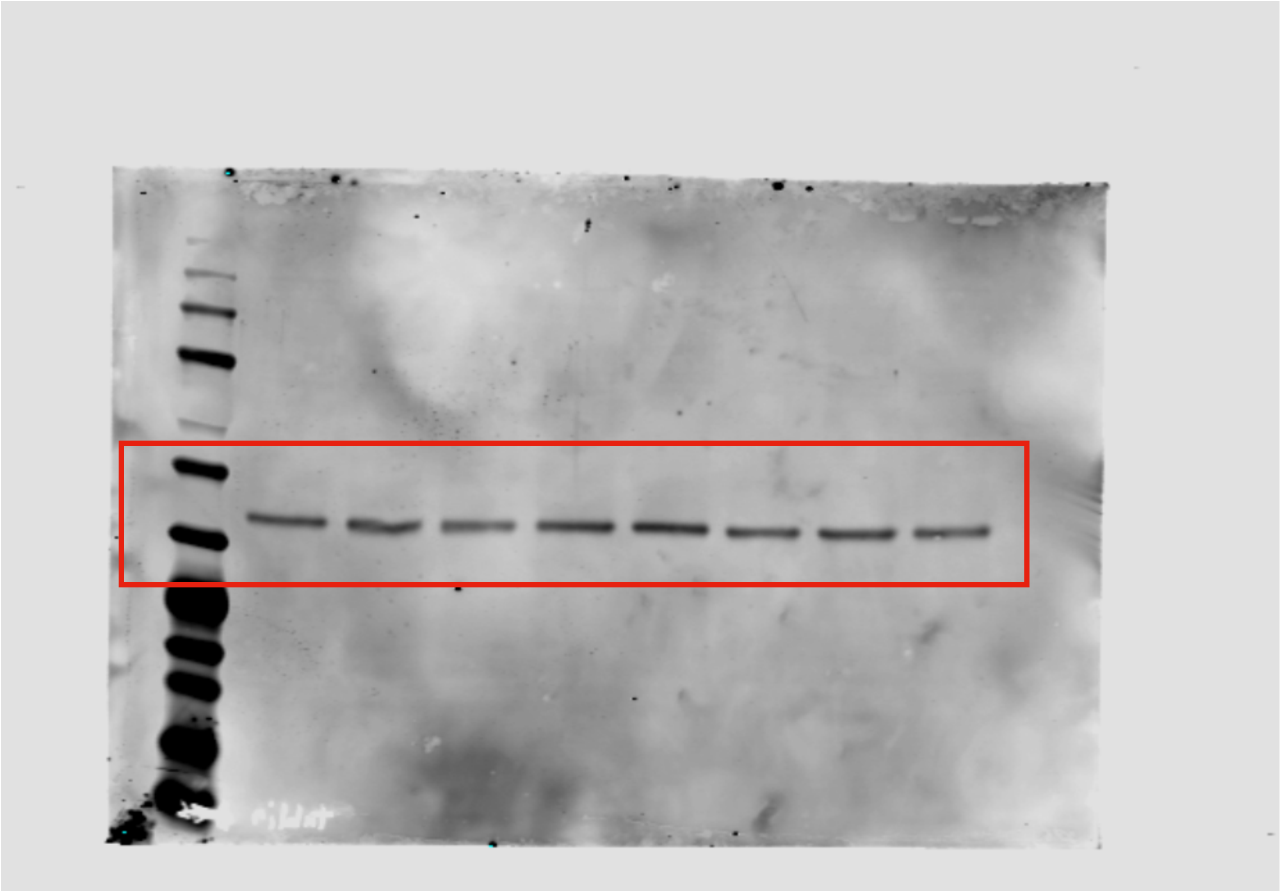

Full unedited gel for Figure 3

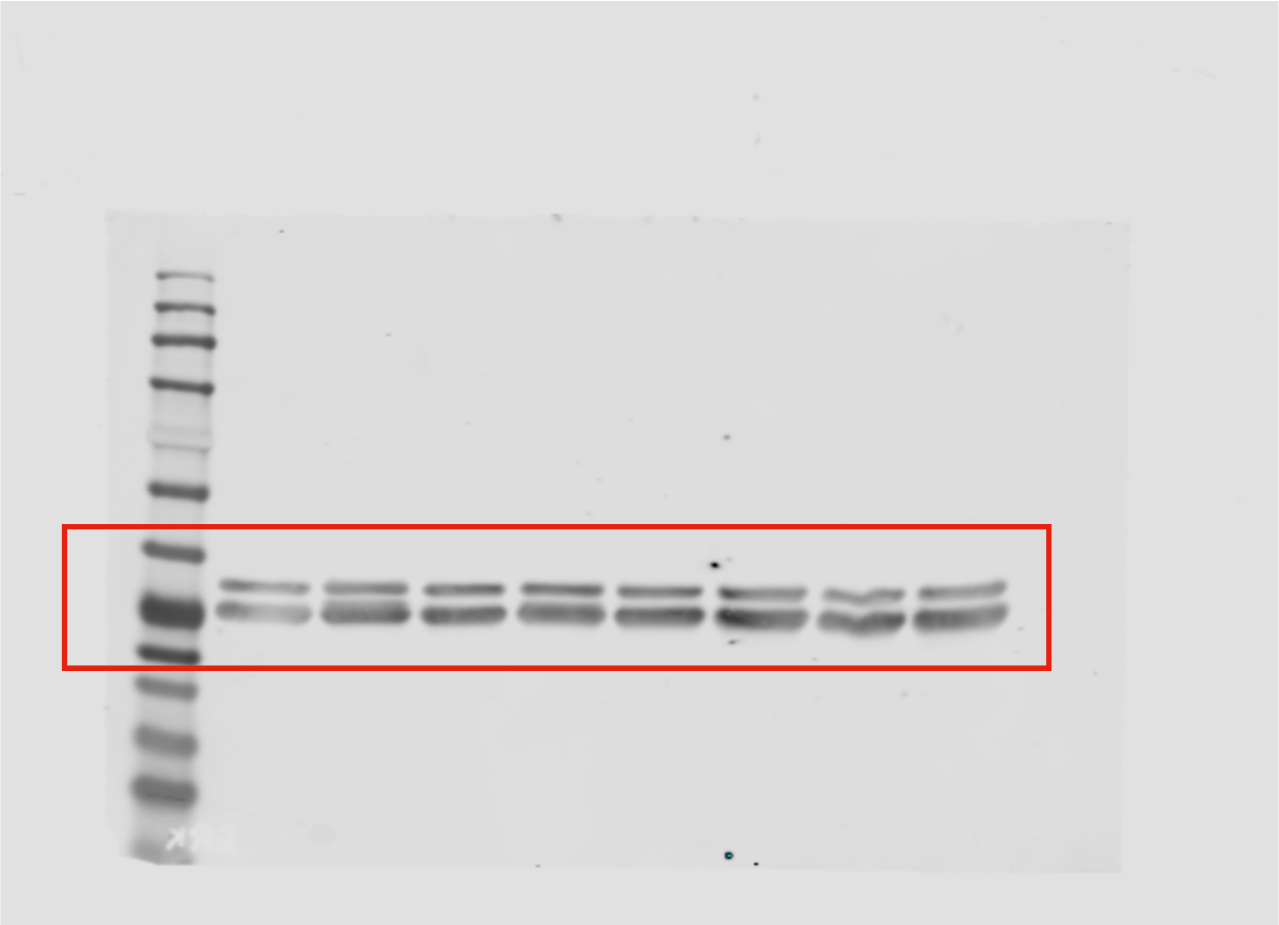

ERK
42 kDa

Full unedited gel for Figure 3

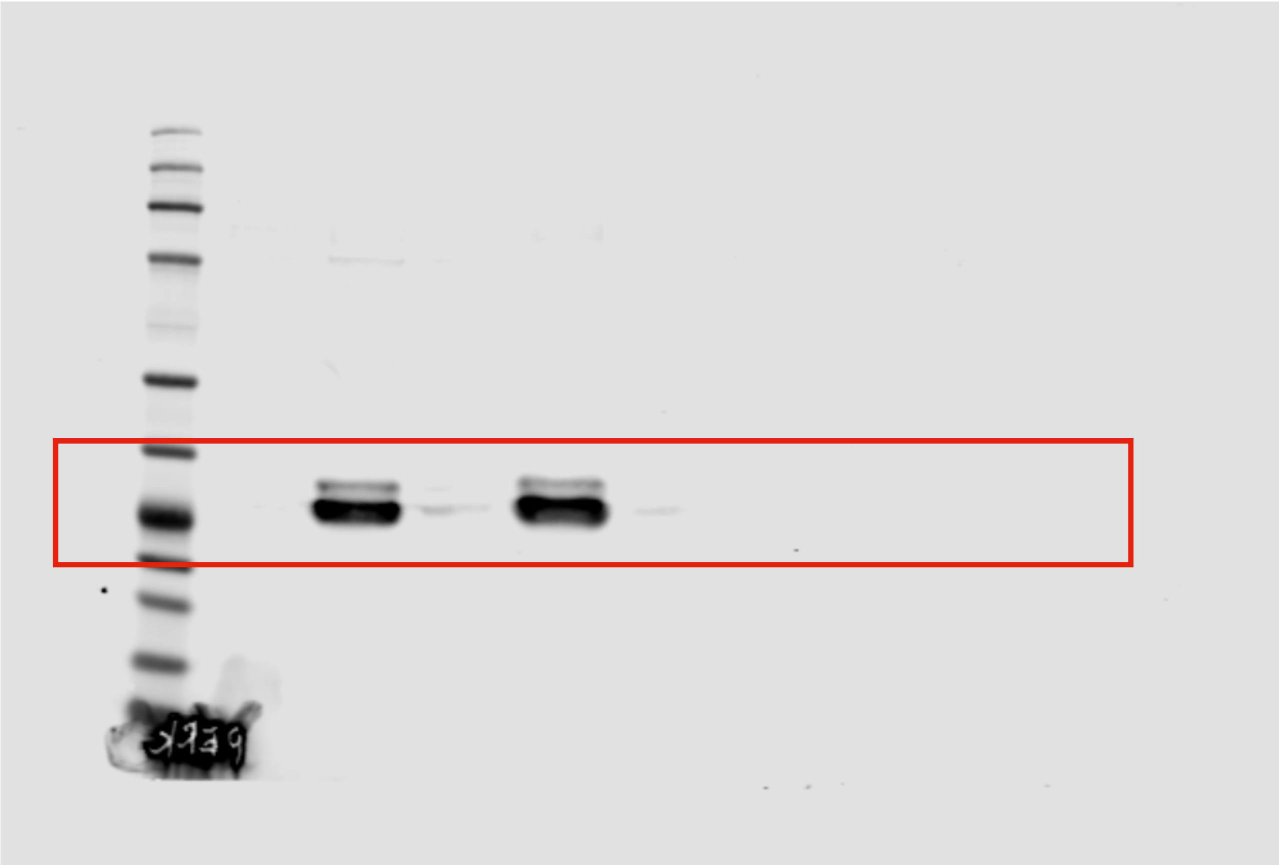

P-ERK
42 kDa

Full unedited gel for Figure 3

RUNX1
55 kDa

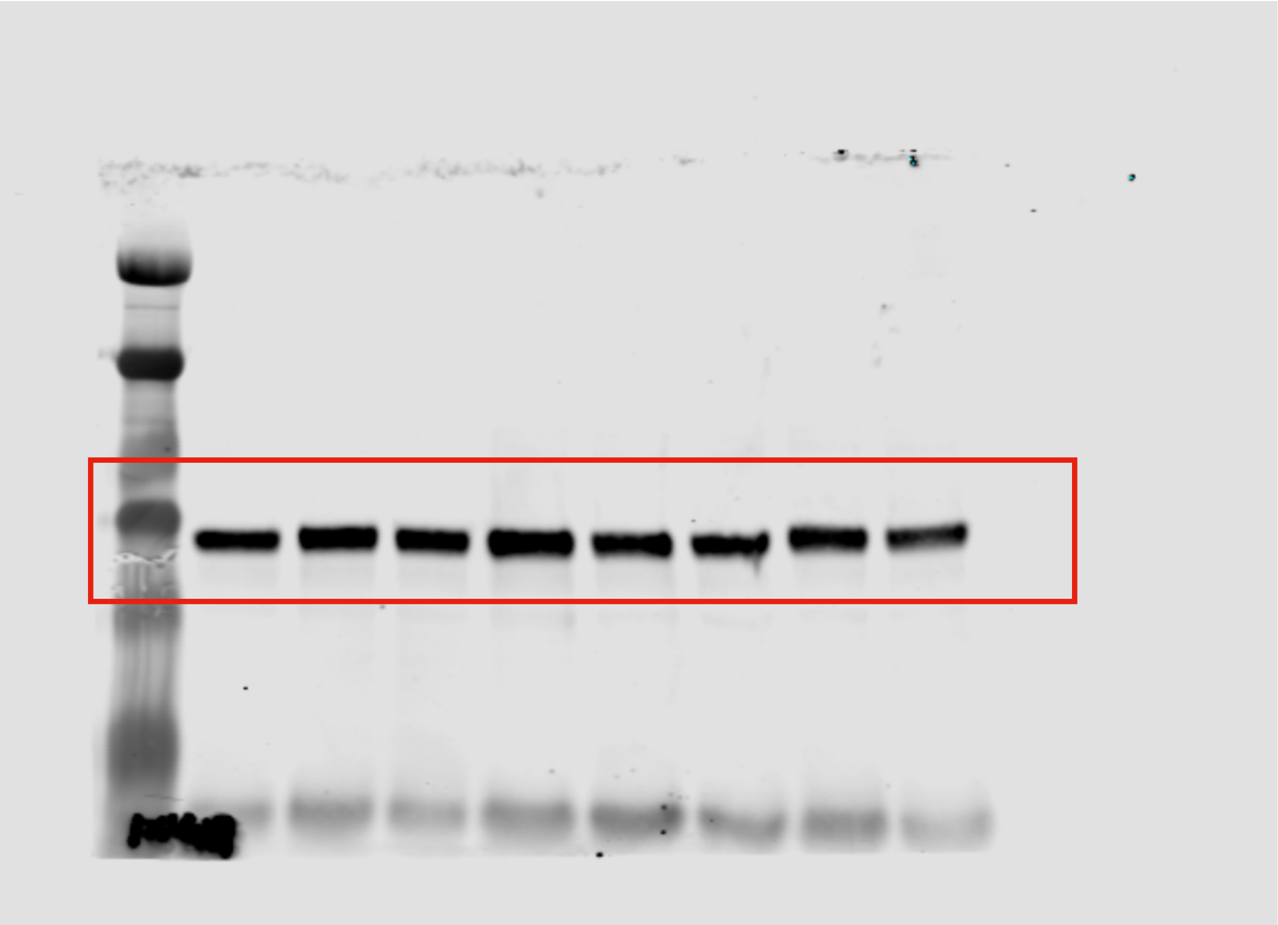

Full unedited gel for Figure 3

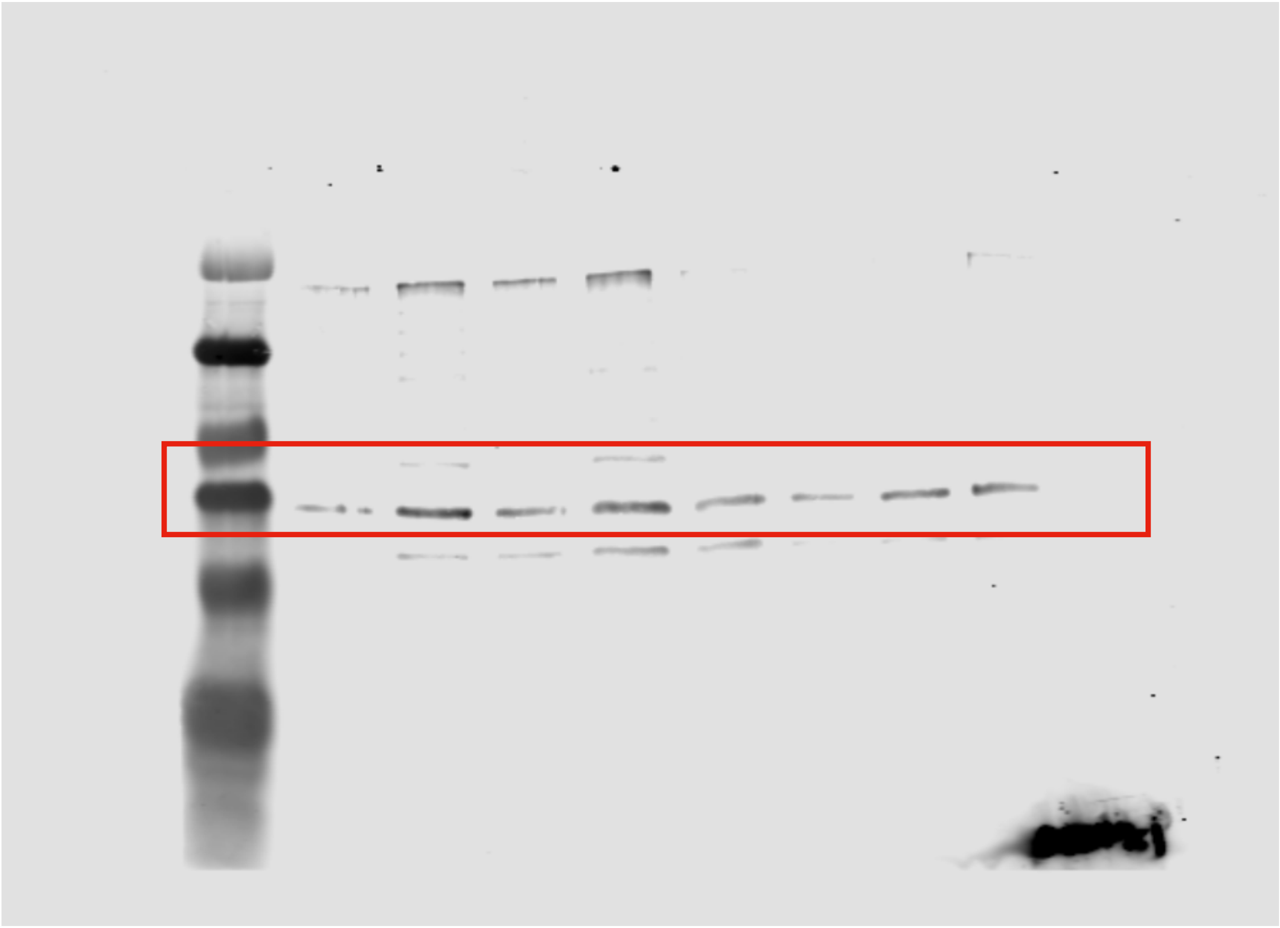

Full unedited gel for supplementary Figure 11

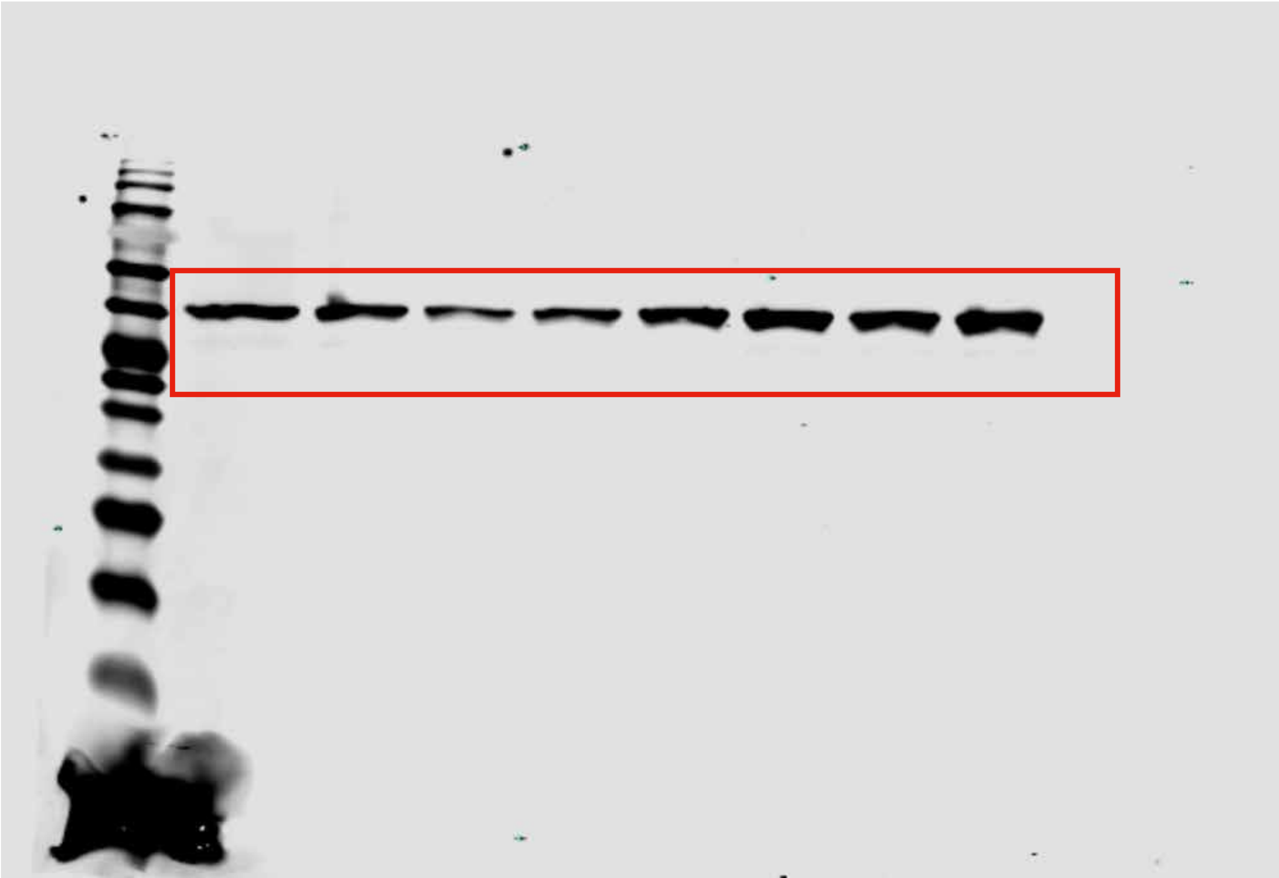

Full unedited gel for supplementary Figure 11

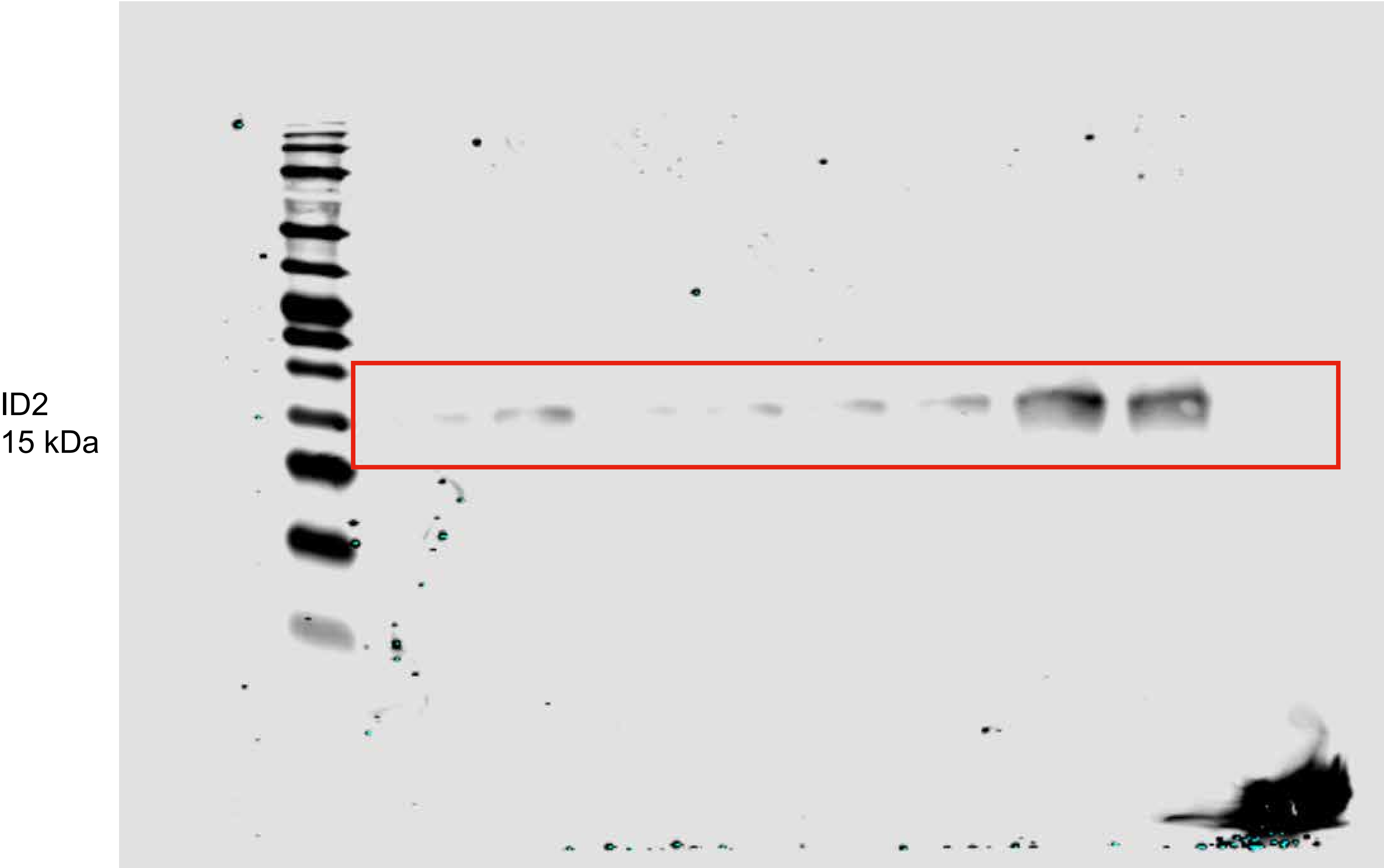

Supplement: Unedited blot and gel images [file jciinsight-10-190240-s013.pdf]
